# Evolution of phenocopying in a dynamical model of developmental trajectories

**DOI:** 10.1371/journal.pcbi.1014385

**Published:** 2026-06-09

**Authors:** Yuuki Matsushita, Archishman Raju

**Affiliations:** National Centre for Biological Sciences, Tata Institute of Fundamental Research, Bangalore, India; Xinjiang Technical Institute of Physics and Chemistry, CHINA

## Abstract

Developmental trajectories are known to be canalized, or robust to both environmental and genetic perturbations. However, even when these trajectories are decanalized by an environmental perturbation outside the range of conditions to which they are robust, they often produce phenotypes similar to known mutants, called phenocopies. This correspondence between the effects of environmental and genetic perturbations has received little theoretical attention. Here, we study an abstract regulatory model that is evolved to follow a specific trajectory. We then study the effects of small and large perturbations to the trajectory, both by changing parameters and by perturbing the state at specific times. We find that the phenomenon of phenocopying emerges in evolved trajectories and is not present in a null model of randomly sampled trajectories. Our results suggest that, in this class of dynamic models, evolution can allow high-dimensional phenotypic landscapes to simultaneously exhibit robustness and phenocopying.

## Author summary

Developmental processes are usually robust, producing reliable outcomes despite environmental perturbations. However, under strong environmental perturbations, organisms can show phenocopies, where phenotypic changes mimic the effects of genetic mutations. This puzzling similarity has been experimentally observed for decades but has received little theoretical attention. Using a computational model of development and evolution, we show that the phenomenon of phenocopying can arise naturally through evolution, even without being directly selected for. Our dynamical systems analysis of the model suggests that evolution results in developmental landscapes where large perturbations push systems toward recognizable mutant-like states. These findings suggest a possible theoretical basis for phenocopying as an emergent property of evolved regulatory landscapes.

## Introduction

A central property of organismal development is canalization, or robustness to both genetic and environmental perturbations. This robustness has been studied both experimentally and theoretically [1–3]. Early experiments by Waddington, Rendel, and others investigated the nature of canalization for different characters in the fly [4–6]. Subsequent studies have found that canalization may be a property of gene regulatory networks [7,8], while others have suggested that so-called capacitors such as Hsp90 may play an important role [9–11]. Computational studies of canalization have focused more on genetic canalization and have shown that robustness can evolve in more abstract regulatory networks [12–15]. Typically, such studies focus on the robustness of the final phenotype to mutations.

However, developmental trajectories often show specific and reproducible outcomes even when environmentally perturbed beyond their normal range of robustness. It has long been known that environmental perturbations can phenocopy known genetic mutants. The term phenocopy was coined by Goldschmidt, who initiated a rich experimental literature on the topic [16–20]. Several different kinds of environmental perturbations have been shown to produce phenocopies, including heat and cold shocks as well as exposure to chemicals such as ether or salts such as silver nitrate. Typically, these perturbations were applied at specific stages of development, were well outside the normal range of fluctuations to which the organism was likely to be exposed, and significantly decreased organismal viability. In *Drosophila*, they produced changes in structures such as bristles, wings, and eyes. Some of these were minor alterations, such as a break in the posterior cross-vein, while others, such as the conversion of halteres into wings, were more dramatic.

The phenomenon of phenocopies suggests that a robust developmental trajectory can be pushed to an alternative trajectory by a sufficiently large perturbation. Furthermore, the experiments demonstrated that the alternative trajectory chosen was highly sensitive to the timing of the perturbation. Indeed, different phenocopies in the fly can be obtained simply by adjusting the timing of a heat shock, as shown in subsequent work [21–24].

In contrast to robustness, phenocopies have received almost no theoretical attention. Fundamentally, this phenomenon indicates that the action of an external environment on the state of the system at particular times can mimic changes to its underlying constituents or parameters. This observation suggests that the developmental landscape has a complex but highly specific structure. It is not known what kinds of properties are required to reproduce this phenomenon.

Waddington used the term “homeorhesis” to describe entire dynamical trajectories that were robust to perturbations during development [25]. Several recent studies have therefore emphasized that development should be understood using concepts from dynamical systems theory [26–28]. The dynamical structure of development is crucial for understanding and interpreting phenocopies for the reasons outlined above.

Recent work has investigated the correspondence between environmental and genetic perturbations. Notably, Ref. [29] examined the effects of environmental and genetic perturbations in a reaction-diffusion and gene regulatory model and found that these effects could be aligned, although such alignment could depend on the timing of the perturbation. Similar alignment of environmental and genetic effects has also been observed in the context of single-celled organisms [30]. Ref. [31] demonstrated how constraints on biological controllers may lead to evolved networks that are robust to both mutational and environmental perturbations. In general, it is not known what kinds of selection pressures could produce such a correspondence. Furthermore, prior studies have tested robustness in the context of endpoints rather than trajectories.

Here, we computationally study an abstract regulatory model evolved to follow a reference trajectory. In particular, we study the following questions in the context of this model: 1) Can phenocopying be obtained as an emergent evolutionary property without direct selection? 2) Do internal and external perturbations have similar effects on an evolved population? To demonstrate the effect of evolution, we compare with a null model that has equivalent functionality to the evolved individuals but is randomly sampled. We use a random sampling method developed in statistical mechanics and computational physics to efficiently sample functional individuals without evolutionary biases [32–34].

The paper is organized as follows. We first describe our dynamical systems model and optimize it to follow a given trajectory. To avoid direct identification with molecular components, we investigate the effects of “internal” and “external” perturbations. The former changes the parameters of the model, whereas the latter changes the trajectory in a timed manner. We describe how the properties of robustness and phenocopying emerge from our evolutionary simulations and end by offering some preliminary explanations for these results from the point of view of dynamical systems theory.

## Model

**Fig 1:**
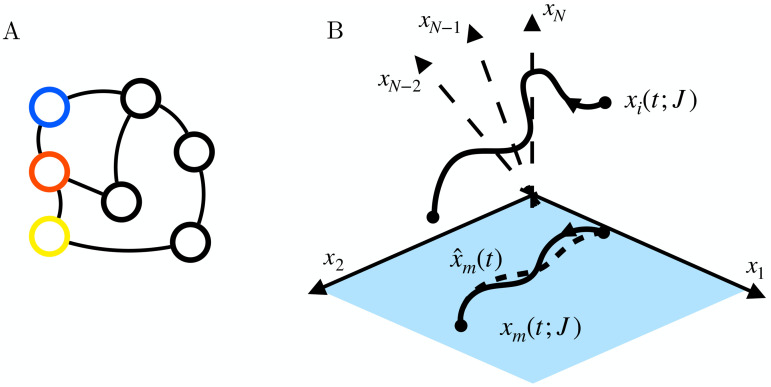
(A) Network model for developmental dynamics. Each node represents the state of the system, and its dynamics is given by Eq (1). Only a subset of the nodes, shown in color, characterize the developmental trajectory. **(B)** A cartoon of the trajectory is shown here. The trajectory $x_{i}(t;J)$ depends on the connections between the nodes *J*, which are inheritable. The developmental process is characterized by $x_{m}(t;J)$, the projection of the trajectory onto an *M*-dimensional subspace (*M* = 2 here for the purposes of visualization). Individuals are evolved to realize a given reference trajectory ${\hat{x}}_{m}(t)$, shown as a dashed line.

Our network model for developmental trajectories is similar to gene regulatory or neural network models previously studied, where the value of each node represents the state of an internal component (Fig 1A) [31,35–40]. We construct a network with *N* nodes, where the dynamics of the state of each node $x_{i}(i=1,2,\cdots ,N)$ is given by a set of ordinary differential equations (ODEs):


$$\begin{array}{c} \frac{dx_{i}}{dt}=F\left(\sum_{j=1}^{N}J_{ij}x_{j}\right)-x_{i}, \end{array}$$ (1)

Here, *F*(*z*) is a sigmoidal function, F(z)=1/{1+exp(−βz)}. We set β to a high value (β=40) to enable switch-like behavior in each node. In Eq (1), the dynamics of the nodes, $x_{i}(t;J)$, is a function of developmental time and a fixed parameter matrix *J*. The elements of the matrix *J* determine the regulatory relations among the nodes. A positive (negative) element *J*_*ij*_ implies an activatory (inhibitory) connection from node *j* to node *i*.

Developmental trajectories are well described by a subset of gene expression states. We therefore characterize the trajectory by a subset of *M* nodes. Mathematically, we denote this subspace of $x_{i}(t;J)$ as $x_{m}(t;J)$, which is the state of a subset of the nodes (m=1,2,⋯,M<N). Hence, in our model, “development” is the generation of a trajectory based on a particular matrix *J*, and “evolution” is the slow dynamics of the matrix elements *J* in a population. The matrix *J* is fixed for an individual on developmental time scales but can vary through evolutionary and random sampling. In the results below, with the exception of S4 Fig, we do not introduce noise into the developmental process.

In the simulations, we set the parameters *N* = 40 and *M* = 3. The main conclusions in this paper are robust to the choice of these parameters, provided that *N* is large enough and *M* is sufficiently smaller than *N*.

Where our model significantly departs from previous such models is in our choice of the fitness function. Rather than optimizing *J* to reach a fixed endpoint, we optimize *J* to follow a desired developmental trajectory (Fig 1B). We first define a reference trajectory ${\hat{x}}_{m}(t)$. The fitness for a given individual is defined as the negative of the distance between the generated trajectory $x_{m}(t;J)$ and the reference trajectory ${\hat{x}}_{m}(t)$. Mathematically,


$$\begin{array}{c} f(J)=-\frac{1}{T}\int_{0}^{T}\sqrt{\sum_{m=1}^{M}{\left(x_{m}(t;J)-{\hat{x}}_{m}(t)\right)}^{2}}dt. \end{array}$$ (2)

More generally, we define the distance between two trajectories $x_{m}^{k}(t)$ and $x_{m}^{l}(t)$, denoted by $D(x_{m}^{k},x_{m}^{l})$, as


$$\begin{array}{c} D\left(x_{m}^{k}(t),x_{m}^{l}(t)\right)=\frac{1}{T}\int_{0}^{T}\sqrt{\sum_{m=1}^{M}{\left(x_{m}^{k}(t)-x_{m}^{l}(t)\right)}^{2}}dt. \end{array}$$ (3)

Our choice of fitness weights different points in the trajectory equally, and this choice is made for simplicity. To maximize *f*(*J*) for a given ${\hat{x}}_{m}(t)$, we performed an evolutionary simulation. We begin with a population of size *L*(=120) with *J* randomly assigned values from {−1,0,1} with probabilities {1/4, 1/2, 1/4}. We calculate the fitness value of each individual based on the generated trajectory. Here, initial conditions for the ODEs are fixed as $x_{m}(t=0)={\hat{x}}_{m}(t=0)$, whereas $x_{i}(t=0)$ (*M* < *i* < *N*) are assigned random values from a uniform distribution on [0, 1]. We do not vary initial conditions during evolutionary and random sampling. After calculating the fitness, individuals are probabilistically selected to form the next-generation population of size *L*. We define the probability of selection for the *l*-th individual with fitness *f*_*l*_ as


$$\begin{array}{c} p(l)=(\exp(\nu \times f_{l}))/Z, \end{array}$$ (4)

$$\begin{array}{c} Z=\sum_{l^{\prime }=1}^{L}\exp(\nu \times f_{l^{\prime }}), \end{array}$$ (5)

where ν=2.0. Mutations were simulated by replacing elements of the matrix *J*, where a given number μ=2 elements from the matrix were replaced with new values randomly assigned as before.

For numerical simulations, we mainly set *L* = 120, μ=2, and ν=2.0. We note that the main results are robust, as long as *L* is large enough, μ is much smaller than the total number of elements in *J* (=*N*^2^), and the selection pressure ν is neither too large nor too small.

To compare the results of our evolutionary simulation, we also sampled functional individuals using a random sampling method. For efficient sampling, we used the multicanonical Monte Carlo (McMC) method to obtain such individuals [32–34] (see S1 Text for more details).

## Results

**Fig 2:**
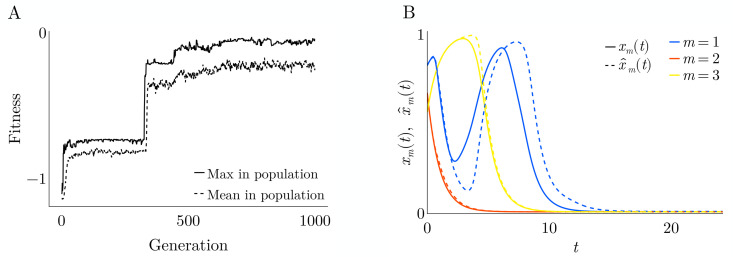
(A) Example of an evolutionary simulation. We plot the maximum and mean fitness values in the population at each generation. **(B)** Time development of $x_{m}(t;J)$ (solid lines) and ${\hat{x}}_{m}(t)$ (dotted lines) for an evolved individual. Different nodes are plotted in different colors.

We conducted our evolutionary simulations and were able to evolve a population whose developmental trajectories closely match the reference trajectory. The value of the fitness as a function of generation is plotted in Fig 2A and shows the increase and eventual saturation of fitness.

We show a typical trajectory of an evolved individual along with the reference trajectory in Fig 2B. We were also able to obtain functional individuals with the desired trajectory from random sampling. We set a threshold fitness value of −0.1 to select functional individuals. We show additional examples of trajectories generated from evolutionary and random sampling, along with their fitness values, in S1 Fig. Here, we note that elementary properties of sampled regulatory networks, such as sparsity and weight fractions, do not depend on the sampling methods we used, but we do not control for other dynamical properties between the two different sampling methods (S2 Fig).

**Fig 3:**
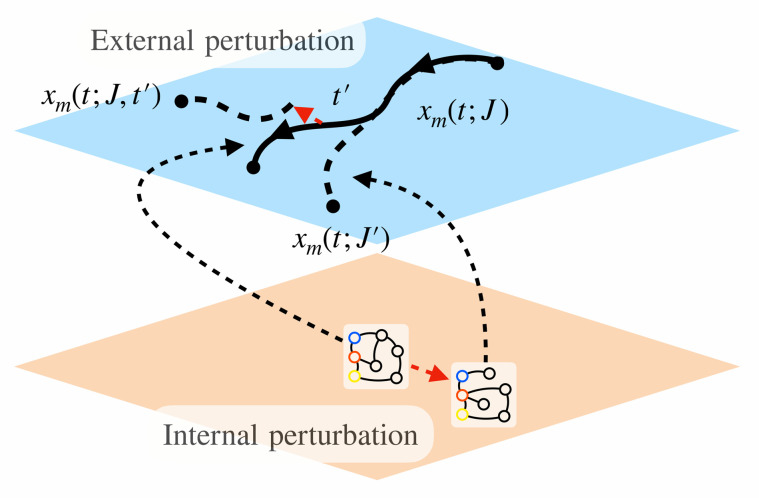
A cartoon of the two kinds of perturbations considered in the paper. $x_{m}(t;J)$ is the base trajectory with given parameters *J*. External perturbations perturb the phenotypic space (like environmental perturbations), whereas internal perturbations perturb the fixed parameter space (like genetic perturbations or mutations).

To investigate whether the trajectories thus evolved were robust against perturbations, we considered two types of perturbations: external and internal (Fig 3). External perturbations (e.g., from the environment) randomly perturb the values of every node, *x*_*i*_, at a fixed time $t^{\prime }$ by values drawn from a uniform distribution between −Δ and Δ. Internal perturbations (e.g., to the genotype) change the matrix *J* and hence affect the trajectory. Given a base trajectory $x_{m}(t;J)$, we denote externally perturbed trajectories as $x_{m}(t;J,t^{\prime })$, where $t^{\prime }$ denotes the time of perturbation. Internally perturbed trajectories are denoted by $x_{m}(t;J^{\prime })$, with a mutated matrix $J^{\prime }$. Internal perturbations are simulated as mutations by replacing $\mu^{\prime }$ elements of *J* with new random values. Unless otherwise specified, we used Δ=0.25 and $\mu^{\prime }=2\;(=\mu )$ for both external and internal perturbations. We did not introduce any state boundaries for the external perturbations Fig 3.

**Fig 4:**
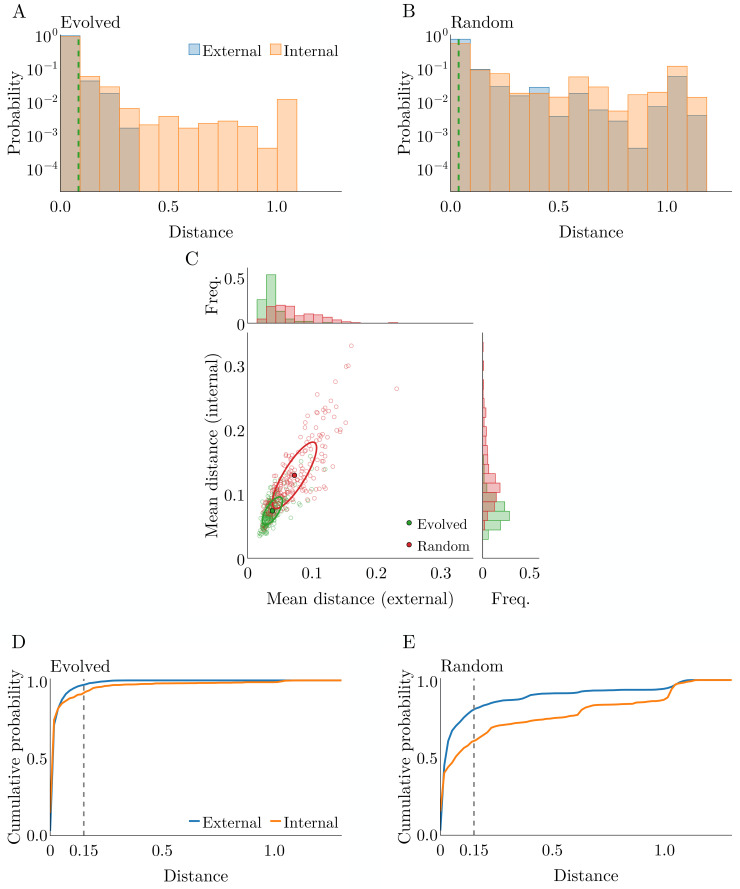
(A-B) Histograms of the distance between the base trajectory and externally perturbed trajectories ($D\left(x_{m}(t;J),x_{m}(t;J,t^{\prime })\right)$, blue) and the distance between the base trajectory and internally perturbed trajectories ($D\left(x_{m}(t;J),x_{m}(t;J^{\prime })\right)$, orange) in an evolved individual (A) and a randomly sampled individual (B). The distance between the base trajectory and the reference trajectory is shown as a dashed vertical line in green (evolved) and red (random). **(C)** Statistical comparison of individuals obtained from evolution and random sampling. To statistically verify the result, we calculate the mean distance of the internally and externally perturbed trajectories from the base trajectory for one individual. Then, we scatter plot the mean distances of 200 individuals from evolution and random sampling. Empty circles represent the mean distances in single individuals, and filled circles represent the mean across 200 individuals obtained by evolution (green) and random sampling (red). Green and red ellipses are drawn according to the covariance matrix (see S1 Text for more details). **(D, E)** Cumulative distribution function (CDF) for distances from the base trajectory in evolved **(D)** and randomly sampled **(E)** individuals.

To investigate the effect of evolution on these trajectories, we compared the effects of perturbations on our evolved population with the effects of perturbations on a randomly sampled population. We generated 5000 externally perturbed trajectories $\left\{x_{m}(t;J,t^{\prime })\right\}$ by perturbing a given trajectory at 50 time points, 100 times at each time point. The time points to be perturbed were chosen by evenly dividing the whole time interval. We also generated 5000 internally perturbed trajectories $\left\{x_{m}(t;J^{\prime })\right\}$. We measured the distance between the perturbed trajectories and the base trajectory $x_{m}(t;J)$. The histograms of distances for evolved and randomly sampled individuals are plotted in Fig 4. Interestingly, evolved individuals exhibit greater robustness, as evidenced by the reduced distance from the base trajectory upon perturbation, for both external and internal perturbations. It should be noted that these individuals were not exposed to any external perturbations during the evolutionary process. The above results are shown for a given individual, but similar effects hold across the population. We see the effects of external and internal perturbations among 200 individuals obtained using evolutionary simulations or random sampling (Fig 4C) and note that evolution canalizes the effects of both external and internal perturbations. These results indicate that the effects of the two different kinds of perturbations are linked, and evolutionary processes selecting for robustness to one kind of perturbation may also produce robustness to the other kind of perturbation.

**Fig 5:**
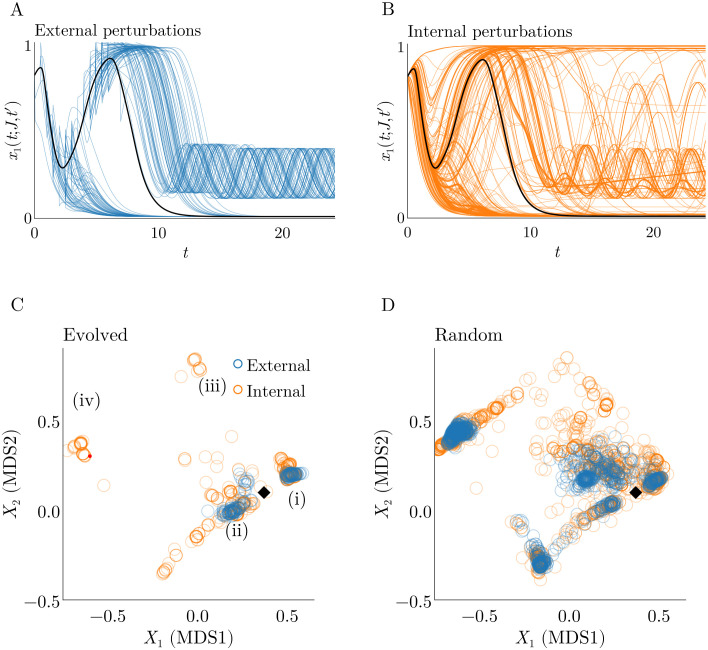
(A-B) The time series of one of the nodes upon (A) externally and (B) internally perturbing an evolved individual. **(C-D)** Two-dimensional MDS mapping of the perturbed trajectories in **(C)** an evolved individual and **(D)** a randomly sampled individual. Each point represents an alternative trajectory obtained either by an external (blue) or internal (orange) perturbation. The black diamond shows the reference trajectory projected onto the space (see S5 Fig for representative trajectories labeled (i) (iv)).

We next examined the structure of trajectories that are sufficiently far from the base trajectory. We note that perturbations with identical strengths can have very different effects in a high-dimensional space when the direction or the point of perturbation is varied. We plot the cumulative distribution function for the distance from the base trajectory for both evolved and randomly sampled individuals in Fig 4D, E. The probability of obtaining a trajectory with a distance greater than a given value is evidently different in evolved and randomly sampled individuals. We set a threshold distance *d* = 0.15 chosen to be larger than the distance between the base and reference trajectories in evolved individuals (typically smaller than 0.1). We note that our filtering leads to differences from two distinct sources: (i) the number of alternative trajectories greater than a given distance is different in the two ensembles, and (ii) the structure of the trajectories conditioned on the distance being greater than a certain threshold distance is different. Our subsequent analysis looked at this structure in detail and is mostly concerned with fractions of trajectories that overlap between internal and external perturbations. Surprisingly, we find the emergence of similar types of alternative trajectories in evolved individuals for both internal and external perturbations with large effects, as shown in Fig 5A and Fig 5B. Although we work with a given reference trajectory, this phenocopying is observed for various choices of reference trajectories and even in the presence of dynamical noise during the developmental process (S3 Fig and S4 Fig).

To visualize the difference between evolved and randomly sampled individuals, we utilized multidimensional scaling (MDS) to dimensionally reduce the trajectories [41]. The basic principle of MDS is to construct a low-dimensional representation that preserves the distance between trajectories as far as possible (see S1 Text for more details). As shown in Fig 5A and B, we projected the trajectories onto the first and second MDS axes, *X*_1_ and *X*_2_. It is clear that the lower-dimensional representation of trajectories in the evolved individual (Fig 5C) is much more tightly clustered than in a randomly sampled individual (Fig 5D). Furthermore, the overlap between external and internal perturbations is much stronger for a typical evolved individual than for a randomly sampled individual. We show some representative alternative trajectories in S5 Fig. We note that this structure of phenocopies is robust to evolutionary parameters, such as population size *L* and mutation rate μ (S6 Fig).

**Fig 6:**
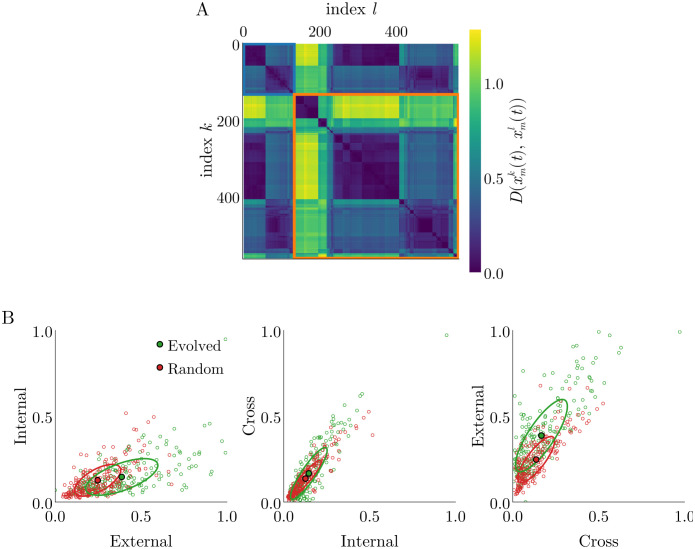
(A) One example of the distance matrix from evolved individuals. We combined the externally and internally perturbed trajectories from one given individual assigned an index to each trajectory. Then, the (*k*, *l*) element of the resulting distance matrix indicates the distance between the trajectories *x*_*k*_ and *x*_*l*_ defined in Eq 3. Two blocks in the matrix shown with a blue (orange) line show pairwise distances among externally (internally) perturbed trajectories with other externally (internally) perturbed trajectories. Non-diagonal blocks indicate pairwise distances among externally and internally perturbed trajectories. For the purposes of visualization, we implemented hierarchical clustering for externally and internally perturbed trajectories separately. **(B)** Similarity between alternative trajectories induced by external and internal perturbations for evolved and randomly sampled individuals. We calculate this mapping using Eq 8. External (Internal) denotes the similarity of alternative trajectories induced by external (internal) perturbations, whereas Cross is the similarity between external and internal perturbations.

To quantify these observations, we calculated pairwise distances between alternative trajectories, defined in Eq 3, as shown in Fig 6. If both trajectories are realized by the same type of perturbation, then closeness implies robustness; if they are realized by different types of perturbations, then closeness implies the presence of phenocopies. Given two sets of trajectories, set 1 with *K* trajectories and set 2 with *L* trajectories, we compute a *K* × *L* distance matrix of pairwise distances between the sets. Each column of this matrix indicates the distance between a point in one set and all points in the other set. We then computed how many elements in the column are less than a threshold *d*_0_, as follows:


$$\begin{array}{c} n_{k}(d_{0})=\sum_{l=1}^{L}1\;\left(0<D_{kl}<d_{0}\right)/L, \end{array}$$ (6)

where *d*_0_ = 0.15. **1**(*A*) is the indicator function defined as


$$\begin{array}{c} 1(A)=\left\{ \begin{array}{c} 1\text{ if }A\text{ is true}\\ 0\text{ otherwise}. \end{array}\right. \end{array}$$ (7)

We quantified the similarity *s* between two sets of trajectories by taking the mean of this column-wise quantity over all rows, thus providing a measure of closeness between the sets. A higher value of this quantity indicates that the two sets are more similar. That is:


$$\begin{array}{c} s=\sum_{k}^{K}n_{k}(d_{0})/K. \end{array}$$ (8)

We note that, when comparing internally or externally perturbed trajectories with themselves (diagonal blocks in Fig 6A), this approach yields identical results regardless of the order of the sets when evaluating overlaps within the same set. On the other hand, when comparing externally perturbed trajectories with internally perturbed trajectories (non-diagonal blocks in Fig 6A), the distance matrix is asymmetric and depends on the order of the sets. In this case, we first computed column-wise values for both the matrix and its transpose. We then averaged the two results to obtain a symmetric quantification. This approach yields identical results regardless of the order of the sets when evaluating overlaps between the two sets. As we show in Fig 6B, self and cross similarities are higher for the evolved population. The localization of the distributions is reflective of the canalization of the trajectories. The overlap between externally and internally perturbed trajectories indicates that the property of phenocopying has evolved even though it is not selected for.

**Fig 7:**
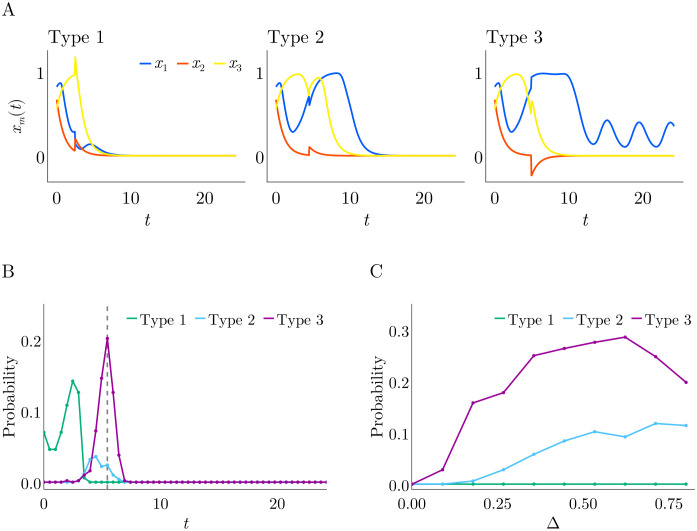
(A) Three representative types of alternative trajectories induced by external perturbation. **(B)** The fraction of perturbations that lead to alternative types of trajectories as a function of the time of perturbation. We tested 500 random external perturbations and classified each trajectory by type. **(C)** The fraction of perturbations that lead to alternative types of trajectories as a function of the strength of the external perturbation. We fixed the perturbation time to the most sensitive point (gray dashed line in B). We tested 500 random external perturbations.

Next, we investigated how changes in the timing and strength of external perturbations give rise to alternative trajectories. By applying hierarchical clustering to the distance matrix for the alternative trajectories obtained in Fig 5A, alternative trajectories can be classified into three distinct trajectory types, as shown in Fig 7A. We then calculated the proportion of alternative trajectories in these distinct trajectory types when the time of perturbation is varied. As shown in Fig 7B, the different types of phenocopies are produced only within specific time intervals, which qualitatively matches prior experimental observations. We also examined how the proportion changes with the strength of the perturbation. As the perturbation strength increases, the proportion of phenocopies rises, although, interestingly, we observed non-monotonic behavior in one case (Fig 7C).

**Fig 8:**
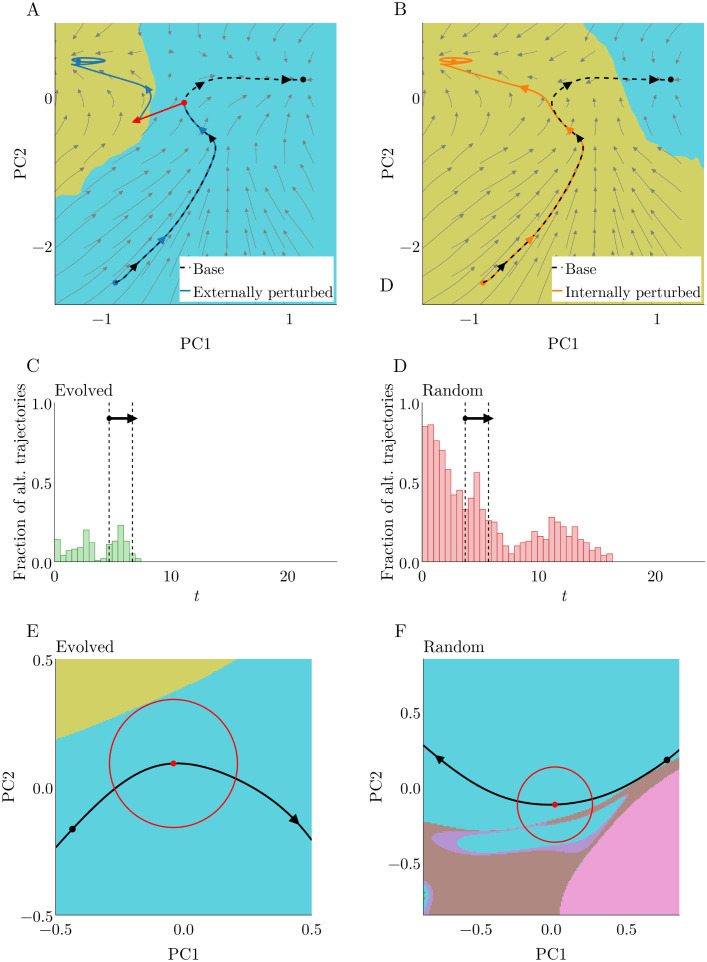
(A-B) Trajectories projected onto their PC axes. The base trajectory is shown as a black dashed line. **(A)** The external perturbation shifts the trajectory, as indicated by the red arrow, leading to a distinct trajectory shown in blue. **(B)** The trajectory obtained as a result of changing the parameters J shifts the basin structure, leading to a trajectory very similar to that in **(A)**, shown in red. **(C-D)** Sensitivity against environmental perturbations as a function of time *t* in an evolved individual and a randomly sampled individual. We apply random external perturbations to the trajectory at each time point 100 times and calculate the fraction of perturbations leading to an alternative trajectory. **(E-F)** Comparison of the phase portrait of an evolved individual and a randomly sampled individual. The red point represents the time point with the peak in Fig 8C and D, and the red circle represents the region into which external perturbations push the trajectory.

To explain these observations, we tried to understand them from a dynamical systems viewpoint. For simplicity, we focused on the structure of the flow near given trajectories and projected the flow onto the principal components (PCs) [42]. In Fig 8A, we plot the flows of a single individual. The basins are also projected onto this space. Note that this is a lower-dimensional projection of a higher-dimensional flow. An external perturbation at a given time can shift the trajectory and take it to an alternative basin. However, the strength of the external perturbation needs to be sufficient to cross the boundaries between the two basins. The trajectory remains robust to small perturbations. In Fig 8B, we see the effect of an internal perturbation, which shifts the basin structure but does not strongly affect the flow.

To compare the phase portrait between an evolved and a randomly sampled individual, we quantified the sensitivity to external perturbations as a function of developmental time. We did this by calculating the fraction of perturbations that lead to an alternative trajectory out of 100 perturbations at each time point. In Fig 8C and Fig 8D, we see that this fraction is strongly suppressed in the evolved case compared to the randomly sampled case at nearly all time points. If we focus on the most sensitive time points and look at the basin structure in the PC space at nearby times, we find that the basin structure of the evolved individual is considerably simpler than that of the randomly sampled individual (Fig 8E and F) in this PC space. While the projected space may not capture the full complexity of the basin structure, it suggests that the differences between evolved and randomly sampled individuals may lie in the difference between their basin structure. Such differences in the basin structure could be observed not only locally near a sensitive point, but also globally along the whole trajectory (S7 Fig). Indeed, the number of attractors in evolved individuals is lower than in randomly sampled individuals (S8 Fig).

Finally, we tested whether our definition of fitness based on the whole trajectory is essential for obtaining phenocopies. If fitness is defined only based on the endpoint, it is difficult to evolve any complex trajectories. Nevertheless, we tested for the presence of phenocopies in populations evolved only to match the endpoint of the reference trajectory. We found that phenocopying can still arise in principle, as shown in S9 Fig, but the number of such phenocopies is greatly reduced. For comparable perturbation strengths, only 34 out of 200 individuals show phenocopies for fitness based on endpoints compared to all individuals showing phenocopies for fitness based on the whole trajectory.

## Discussion

We have numerically demonstrated that the phenomenon of phenocopying can emerge with an abstract evolutionary model of developmental systems. To investigate this phenomenon, we evolved whole developmental trajectories rather than a fixed endpoint phenotype. Therefore, our fitness is a function of the whole trajectory rather than simply the final phenotype. Such dynamic fitness is usually accounted for using age or stage dependence. We make the simplifying assumption that the whole trajectory is weighted equally when calculating the fitness. We also tested that trajectories evolved with a fitness based on the endpoint lead only very infrequently to phenocopies. More work is needed to understand how different definitions of the fitness along the trajectory change the structure of phenocopies.

By explicitly accounting for perturbations that could be internal (e.g., genetic) or external (environmental), we show that evolving for robustness to internal perturbations can automatically create robustness to external perturbations. A limitation of our study is that external perturbations are modeled as global and unstructured. It may be possible that biological perturbations are localized and structured. Alternatively, perturbations in classic experiments, such as heat shocks, may have global effects. Our aim here is to probe the effect of external perturbations in the simplest possible way and not to model biological complexity. Nevertheless, it would be interesting to study if our results agree in cases which are more structured and what kinds of perturbations are likely to lead to phenocopies.

Our simulation results are in accordance with the hypothesized single mode of canalization, suggesting that the strongly canalized parts of developmental trajectories will be robust to both environmental and genetic perturbations [43]. However, we note that this “mode” may not be a single molecule and could be context dependent [44]. Our work contributes to the literature on how the effects of environmental and genetic perturbations may be related by investigating it in an explicitly dynamic scenario.

In the present work, we demonstrated that phenocopying can emerge as a byproduct of evolutionary robustness by comparing with randomly sampled individuals as a null model. We have preliminarily shown that our main result is robust against changes in evolutionary parameters or the addition of developmental noise, but the details of phenocopies could depend on such parameters. Further theoretical studies are needed to investigate this dependence.

The phenomenon of phenocopies has a large experimental literature dating back to the early days of embryology. However, it has received little theoretical attention. In our framework, we define phenocopies as alternative developmental trajectories which can be produced both by large external perturbations to the state of a system and by changing internal parameters. We find that these alternative trajectories emerge from our evolutionary simulations without being selected for. Our simulations evolved the system to follow a fixed reference trajectory. When we condition on trajectories being larger than a given threshold distance, evolved individuals show the property of phenocopying but randomly sampled populations do not. This general idea of phenocopies as similar effects resulting from large external and internal perturbations may have applications in other very different contexts [45–48].

Prior experimental work demonstrated that phenocopies depend sensitively on the timing and strength of the external perturbation. Very large perturbations would produce unviable individuals. We find sensitive dependence on timing but have no concept of unviability in our model. Furthermore, our model is abstract, but the correspondence we observe could also be tested for more realistic gene regulatory models [49].

More modern experimental work is needed to understand phenocopies. A recent study suggested that general stress-response mechanisms may be responsible for phenocopies in the case of the bithorax phenocopy induced by ether or heat stress [50,51]. In general, however, little data exist comparing gene expression pattern changes under environmental perturbations in developmental systems. Another study observed that applying directional selection on wing size in flies decanalizes the system simultaneously to both genetic and environmental perturbations, which is consistent with our results showing a correspondence between the effects of the two kinds of perturbations [52].

Waddington’s experiments on genetic assimilation, which have recently been repeated and studied [53–55], relied on the presence of phenocopies. His landscape was essential in providing an explanation for genetic assimilation. A recent study theoretically studied genetic assimilation but assumed perfect canalization of both the base and perturbed trajectories, which is not true in general [56]. Therefore, our work may provide further insight into the phenomenon of genetic assimilation, which has found applications across species [57,58].

In the study of robustness and plasticity, the dynamical viewpoint of development can be very useful. However, since developmental trajectories are in high-dimensional space, the structure of their phase space can be quite complex [59]. Some have suggested that linearizing the dynamics and looking at the structure of the modes may provide insight into why genetic and environmental perturbations have similar effects because they are mediated through “soft modes” [47]. However, the phenomenon of phenocopies involves large perturbations and is inherently nonlinear. Although it is more difficult to provide general results in this case, our numerical results obtained using dimensional reduction suggest that this phenomenon might emerge because evolution simplifies the complex basin structure of high-dimensional systems. This is reminiscent of the low-dimensional structure of high-dimensional systems observed in other contexts and might be regulated by “slow variables”, as in [60]. Further theoretical studies are required to understand this.

## Supporting information

S1 Text Supplementary materials and methods. (PDF)

S1 FigExamples of trajectories $x_{m}(t;J)$ obtained from evolutionary sampling (top) and random sampling (bottom), along with their fitness.(TIF)

S2 Fig Comparison of the sparsity (A) and the fraction of the weights −1, 0, and 1 in the sampled regulatory matrix *J* (B) from evolutionary sampling (green) and random sampling (red). (TIF)

S3 FigComparison of alternative trajectories induced by external and internal perturbations using various reference trajectories.Each row represents a case with a different reference trajectory ${\hat{x}}_{m}(t)$. (Left column) Reference trajectory and original trajectory obtained from an evolved individual, as in Fig 2B. Alternative trajectories induced by external perturbations (center column) and internal perturbations (right column), as in Fig 5A and 5B. Reference trajectory 1 is the case of a trajectory immediately directed to a final state. This case is the simplest and close to endpoint-based fitness rather than trajectory-based fitness. Reference trajectory 2 has monotonically decreasing *x*_*m*_ values, albeit at different times. Reference trajectory 3 shows sequential excitable behavior but eventually converges to a final state. Only the dynamics of a single node is shown when plotting the internally and externally perturbed trajectories.(TIF)

S4 Fig Comparison of alternative trajectories induced by external and internal perturbations under developmental noise. See S1 Text for details of the simulation.
(TIF)

S5 Fig Representative trajectories at each point (i)–(iv) in Fig 5C and Fig 5D. (TIF)

S6 FigMDS mapping with different parameters in evolutionary sampling.We sampled individuals with different evolutionary parameters. Then, we tested external and internal perturbation experiments with Δ=0.25 and $\mu^{\prime }=2$. (Top) We changed the population size to twice the original size in the main paper (i.e., *L* = 240). (Bottom) We changed the mutation size to 4 from the original size in the main paper (i.e., μ=4). In both cases, overlap of the two distributions is observed.(TIF)

S7 Fig All alternative trajectories induced by external perturbation in an evolved individual (A) and a randomly sampled individual (B) in Fig 8E and 8F. Different colors indicate trajectories that reach different attractors. Trajectories are analyzed by hierarchical clustering according to pairwise distance, and trajectories in the same cluster are shown in the same color.
(TIF)

S8 Fig Comparison of the number of attractors between evolved individuals and randomly sampled individuals. The fraction of individuals with more than five attractors is represented as a single bar labeled 5 < .
(TIF)

S9 Fig The evolved trajectory of an individual, along with internally and externally perturbed trajectories when fitness is based on the endpoint. (TIF)
