# Supplementary material for: Evolution of phenocopying in a dynamical model of developmental trajectories
Source: PLoS Comput Biol. 2026 Jun 9;22(6):e1014385. doi: 10.1371/journal.pcbi.1014385 (PMC13262954; doi:10.1371/journal.pcbi.1014385)
Supplement: S1 Text — (PDF) [file pcbi.1014385.s001.pdf]

S1 Text. Supplementary Materials and Methods

Sampling from evolutionary simulation

We sampled individuals from evolutionary simulations with different evolutionary histories. In a single evolutionary simulation, we iterated a unit evolutionary process (mutation, calculation of fitness, and selection) for 1000 generations. We assembled 200 independent evolutionary histories in which at least one individual reached a fitness larger than -0.1. At the final generation of each evolutionary simulation, we randomly sampled one individual whose fitness was larger than -0.1.

Random sampling method

We performed random sampling of functional individuals by multicanonical Monte Carlo (McMC) simulation. In general, functional individuals are too rare to sample independently. McMC makes this sampling possible by uniformly sampling the evaluation function (fitness). We perform a Monte Carlo simulation in which the inverse of the number of states is utilized as transition weights to realize uniform sampling for a given evaluation function. That is, the transition rate from the state with evaluated value (fitness) f_1 to that with f_2 is set as

$$p(f_1 \to f_2) = \min\left[\frac{g(f_1)}{g(f_2)}, 1\right], \tag{1}$$

where $g(f)$ is the number of states (genotypes here) whose fitness values are f. One Monte Carlo step is performed as a mutation in the evolutionary simulation.

In general, we cannot obtain the number of states as a function of fitness. However, it is well known that a rough estimation works sufficiently well for uniform sampling. To implement this numerical sampling, we used the Wang-Landau learning algorithm for this estimation [1,2], following a previous study [3]. In the Wang-Landau method, all of the $g(f)$ are initially set to 1; that is, each f is sampled equally. Throughout the sampling process, these $g(f)$ are updated as $g(f) \to C \times g(f)$ when the corresponding f is visited in a Monte Carlo update. By this update rule, a fitness value more (less) likely to be visited becomes less (more) likely to be sampled in the future. This allows the fitness to be sampled effectively uniformly. In the algorithm, the factor C is also updated on a longer time scale. We set the initial $C = C_0 = e$ and updated C as $C \to \sqrt{C}$. In practice, we discretized $g(f)$ into a histogram with 40 bins from $-\sqrt{M}$ to 0. We simulated 2×10^6 Monte Carlo steps with a fixed update factor C and repeated this 20 times while updating C each time. The fitness as a function of genotype is calculated as in our evolutionary simulation (Eq (2), Main Text).

After updating $g(f)$ as described above, we sampled individuals uniformly in fitness by utilizing the obtained $g(f)$ and choosing functional individuals. We chose 200 individuals whose fitness was larger than -0.1. To avoid correlations between individuals, we sampled them with a sampling interval. We therefore skipped 120 functional individuals before choosing another functional individual from the sample.

MDS and PCA mapping of trajectories

We evaluated the relation between alternative trajectories by classical multidimensional scaling (MDS), as in Fig 5C and 5D. For this, we calculated pairwise distances for all pairs of alternative trajectories as in Eq (3) and used the Julia package MultivariateStats.jl for MDS. To avoid scale differences, we created an identical MDS mapping by combining all the data points (alternative trajectories) from evolved and randomly sampled individuals and calculating the pairwise distance matrix for them.

In Fig 8A and 8B, we used PCA to explore how the phenomenon of phenocopying is realized in an evolved individual. Then, we performed PCA for a representative trajectory obtained from an evolved individual, the original trajectory $x_i(t; J)$, and one alternative trajectory (either $x_i(t; J, t')$ or $x_i(t; J')$).

For each of Fig 8E and 8F, we performed PCA on data points taken from each of the trajectories. We picked up the part of the trajectory $\{x_i(t)\}$ around the most sensitive point to visualize the dimensionally reduced basin structure close to the sensitive point (the actual time interval taken is shown in Fig 8C and 8D).

S7 Fig shows the basin structure close to the trajectory in an evolved and a randomly sampled individual. For this, we solved ODEs with initial conditions near the trajectory and then performed

PCA on all of the data points in the representative trajectories with different final states, in an evolved individual (A) and a randomly sampled individual (B). We then plotted all the alternative trajectories in this PCA space. We note that, in this case, two dimensions are not sufficient to observe the basin structure clearly, as some basin structures overlap in S7 Fig.

Calculating the number of attractors

For further comparison of the structure between evolved and randomly sampled individuals, we measured the number of attractors for each individual. For a given individual, we prepared 10^4 random initial conditions generated from a uniform distribution ranging from 0 to 1. We then solved the ODEs starting from these initial conditions for a sufficient time (from $t = 0$ to $t = 100$) and found the attractors. We found that the typical time to converge to an attractor was less than 50. We note that the attractors could be fixed points, limit cycles, or chaotic. We classify stable fixed points by using the Julia package DiffEqCallbacks, which classifies steady states by checking that the time series converges to a steady state. We used a tolerance of 10^{-5}. We classify stable fixed points as different if the distance (standard norm) between them is greater than 10^{-1}. If the attractor does not reach a stable fixed point by time $t = 100$, we solve the ODE further using the state at $t = 100$ as an initial condition. We stop the evolution after we reach a point that is close to the point at $t = 100$ with a tolerance of 10^{-1}. To distinguish these attractor sets, we used the following procedure. Suppose two attractor sets are obtained after time evolution from different initial conditions. We chose the point at $t = 100$ from the second set and compared it with the subsequent time evolution of the first set. If any point in the first set was found to be at a distance (standard norm) less than 10^{-1} from the point at $t = 100$ in the second set, we classified the two attractors as equivalent. This procedure may overestimate the number of attractors in the case of chaotic attractors that are slow to return to the initial point. Such chaotic attractors are rare in our system, and we did not classify the attractor number beyond 5 to avoid such overestimates.

Evolutionary simulation with dynamical noise

For the evolutionary simulation with dynamical noise, we changed Eq (1) in the main text to the following Langevin equation:

$$\frac{dx_i}{dt} = F\left(\sum_{j=1}^{N} J_{ij}x_j\right) - x_i + \sigma\eta_i(t), \tag{2}$$

where the last term is the stochastic term. The stochastic term was introduced as additive noise, where σ is the noise amplitude and $\eta_i(t)$ is an independent Gaussian white noise process satisfying

$$\langle \eta_i(t) \rangle = 0 \tag{3}$$

$$\langle \eta_i(t)\eta_j(t') \rangle = \delta_{ij}\delta(t - t'). \tag{4}$$

We set $\sigma = 0.025$ and used the Euler-Maruyama method with time step $\Delta t = 5 \times 10^{-2}$ for the numerical simulation.

Evolutionary simulation with endpoint-based fitness function

As shown in Eq (2) in the main text, the fitness function in the main text evaluates how close the generated trajectory is to the reference trajectory at each time point, i.e., it is trajectory-based. In contrast, we can consider an endpoint-based fitness function that only evaluates proximity at the endpoint. We can modify the fitness function in the following way:

$$f(J) = -\sqrt{\sum_{m=1}^{M} (x_m(T; J) - \hat{x}_m(T))^2}, \tag{5}$$

where T is the final time point. The rest of the procedure for finding phenocopies remains the same.

References

[1] F Wang and D P Landau. Determining the density of states for classical statistical models: a random walk algorithm to produce a flat histogram. *Phys. Rev. E Stat. Nonlin. Soft Matter Phys.*, 64(5 Pt 2):056101, November 2001.

[2] F Wang and D P Landau. Efficient, multiple-range random walk algorithm to calculate the density of states. *Phys. Rev. Lett.*, 86(10):2050–2053, March 2001.

[3] Shintaro Nagata and Macoto Kikuchi. Emergence of cooperative bistability and robustness of gene regulatory networks. *PLoS Comput. Biol.*, 16(6):e1007969, June 2020.
