# Supplementary figures and images for: Evolution of phenocopying in a dynamical model of developmental trajectories
Source: PLoS Comput Biol. 2026 Jun 9;22(6):e1014385. doi: 10.1371/journal.pcbi.1014385 (PMC13262954; doi:10.1371/journal.pcbi.1014385)

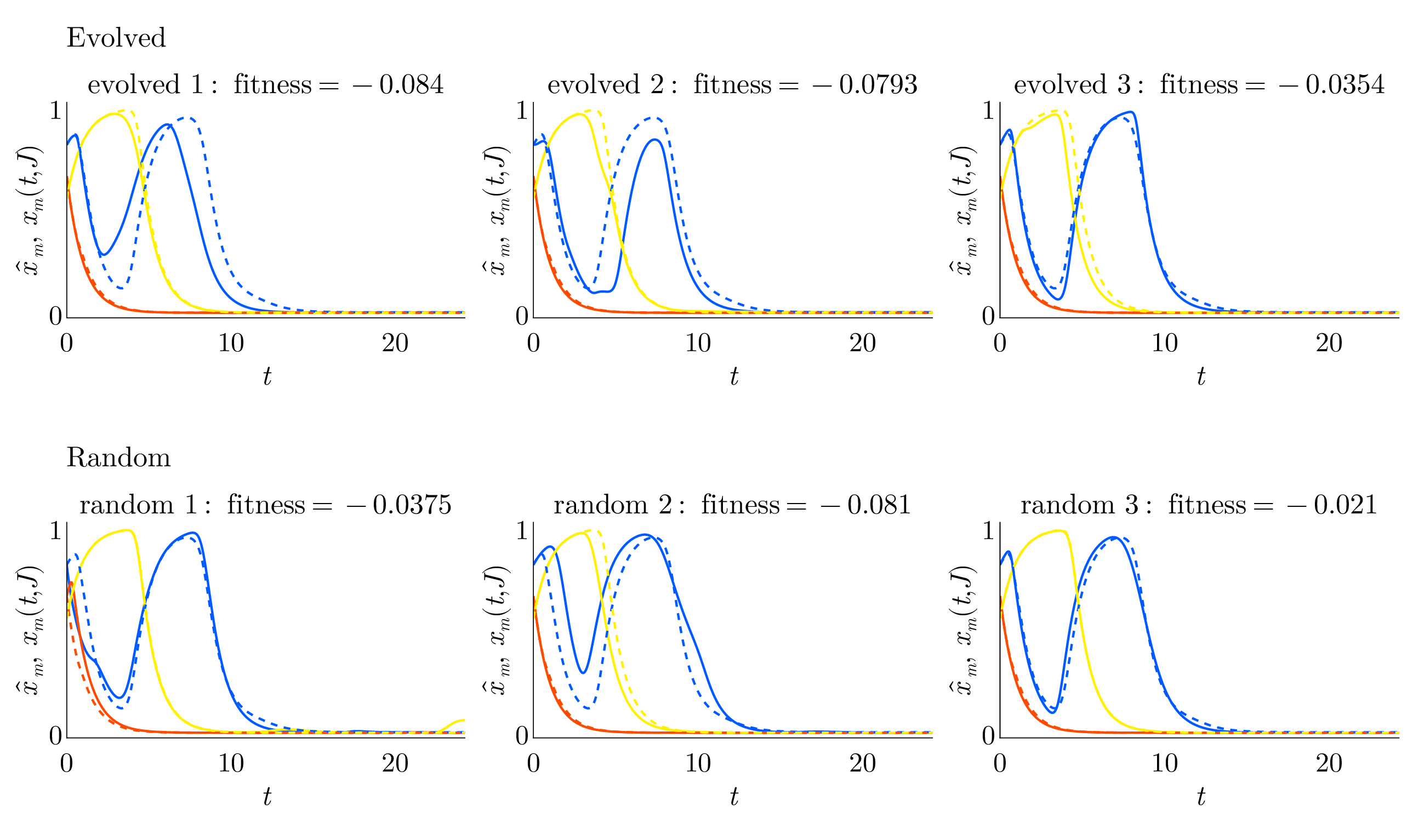

Supplement: S1 Fig — (TIF) [file pcbi.1014385.s002.tif]

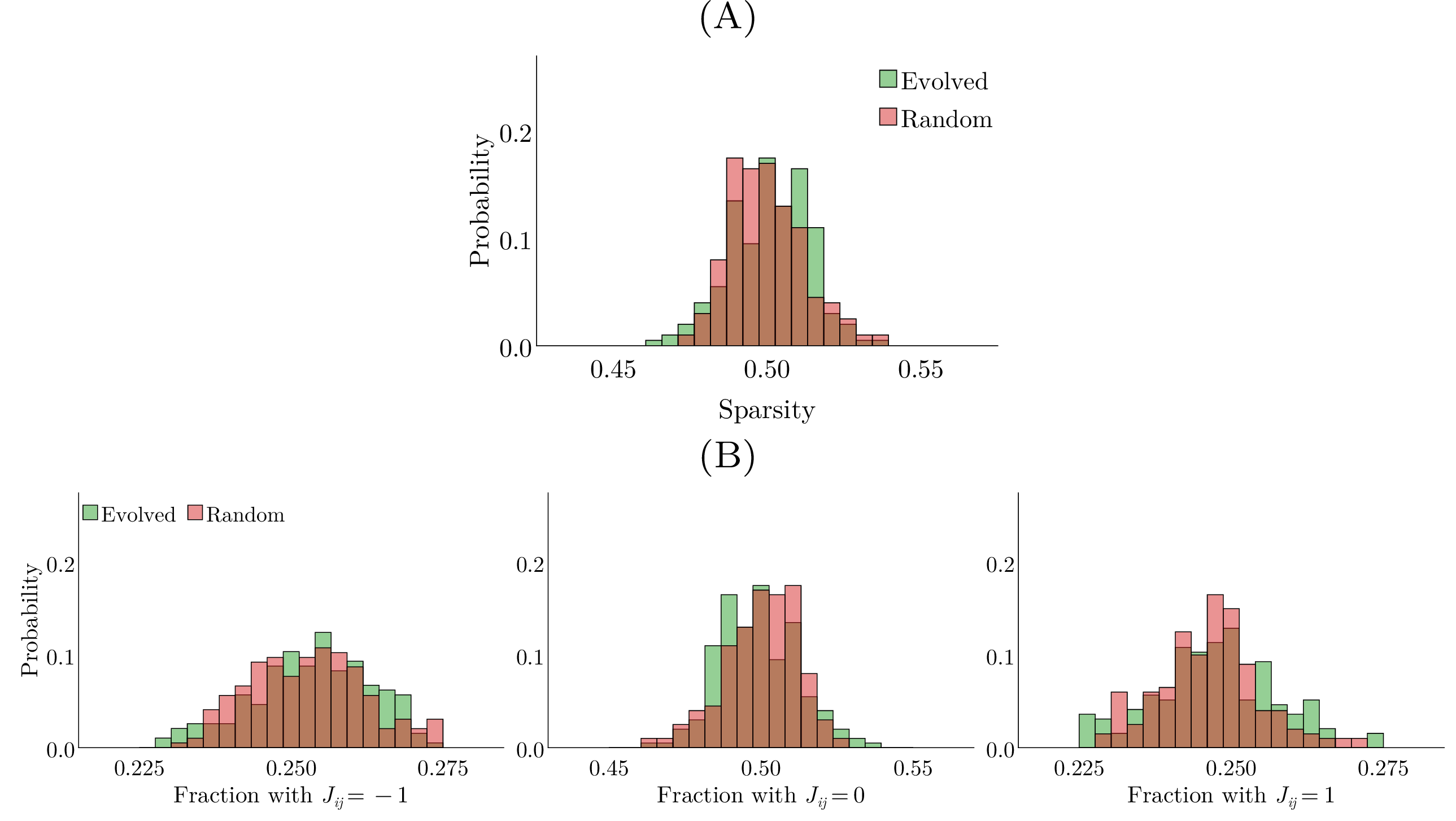

Supplement: S2 Fig — (TIF) [file pcbi.1014385.s003.tif]

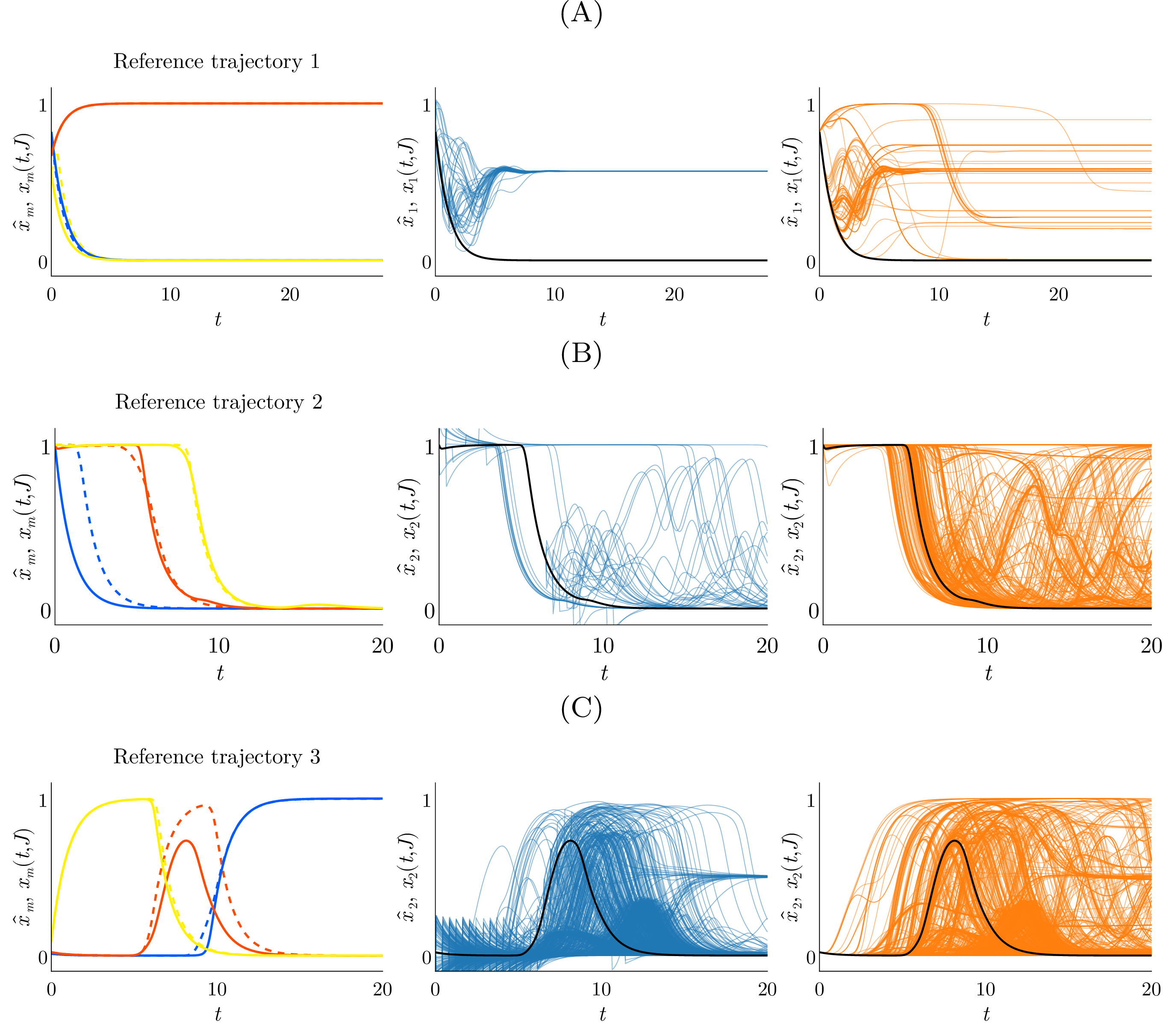

Supplement: S3 Fig — Each row represents a case with a different reference trajectory x^m(t). (Left column) Reference trajectory and original trajectory obtained from an evolved individual, as in Fig 2B. Alternative trajectories induced by external perturbations (center column) and internal perturbations (right column), as in Fig 5A and 5B. Reference trajectory 1 is the case of a trajectory immediately directed to a final state. This case is the simplest and close to endpoint-based fitness rather than trajectory-based fitness. Reference trajectory 2 has monotonically decreasing xm values, albeit at different times. Reference trajectory 3 shows sequential excitable behavior but eventually converges to a final state. Only the dynamics of a single node is shown when plotting the internally and externally perturbed trajectories. (TIF) [file pcbi.1014385.s004.tif]

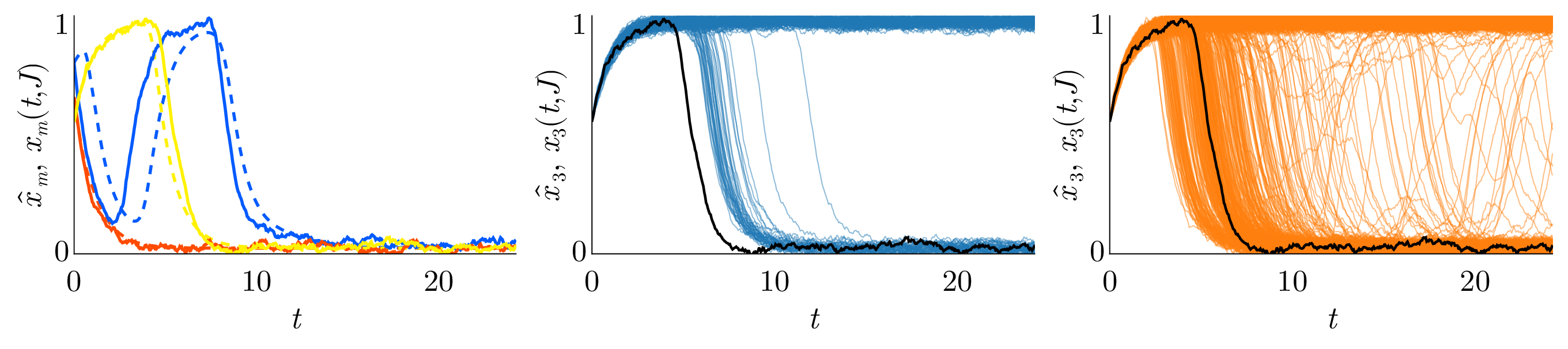

Supplement: S4 Fig — See S1 Text for details of the simulation. (TIF) [file pcbi.1014385.s005.tif]

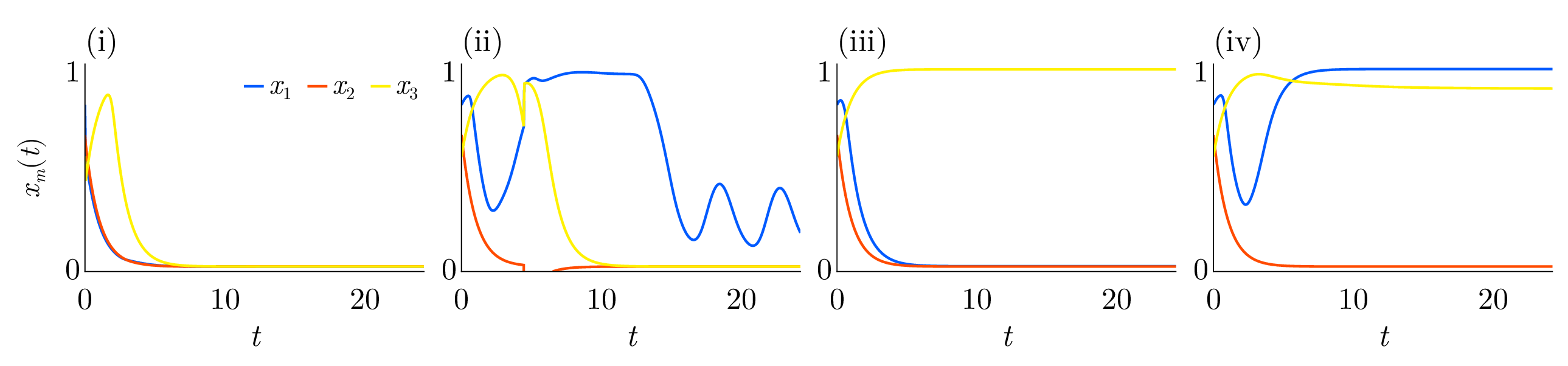

Supplement: S5 Fig — (TIF) [file pcbi.1014385.s006.tif]

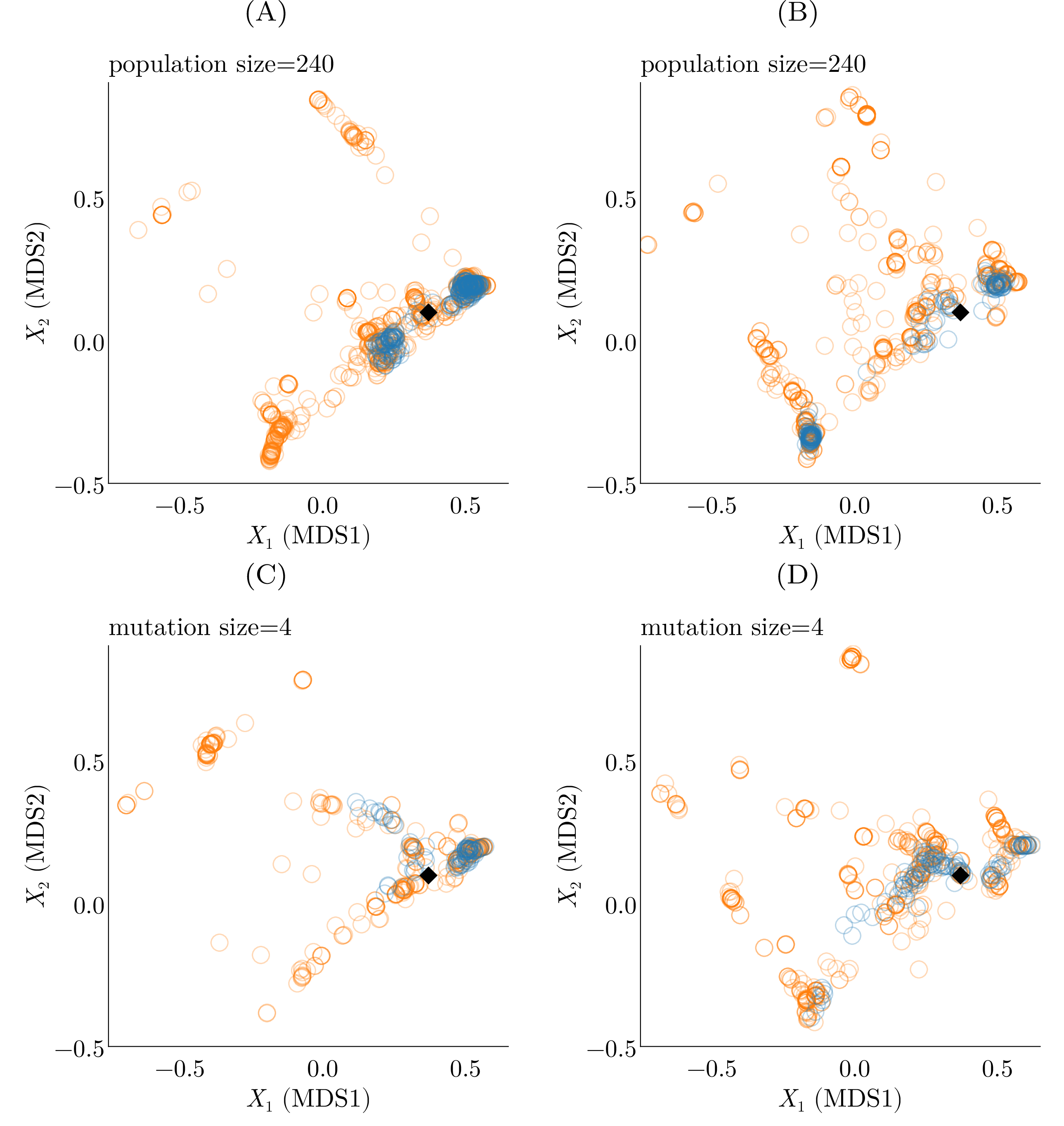

Supplement: S6 Fig — We sampled individuals with different evolutionary parameters. Then, we tested external and internal perturbation experiments with Δ=0.25 and μ′=2. (Top) We changed the population size to twice the original size in the main paper (i.e., L = 240). (Bottom) We changed the mutation size to 4 from the original size in the main paper (i.e., μ=4). In both cases, overlap of the two distributions is observed. (TIF) [file pcbi.1014385.s007.tif]

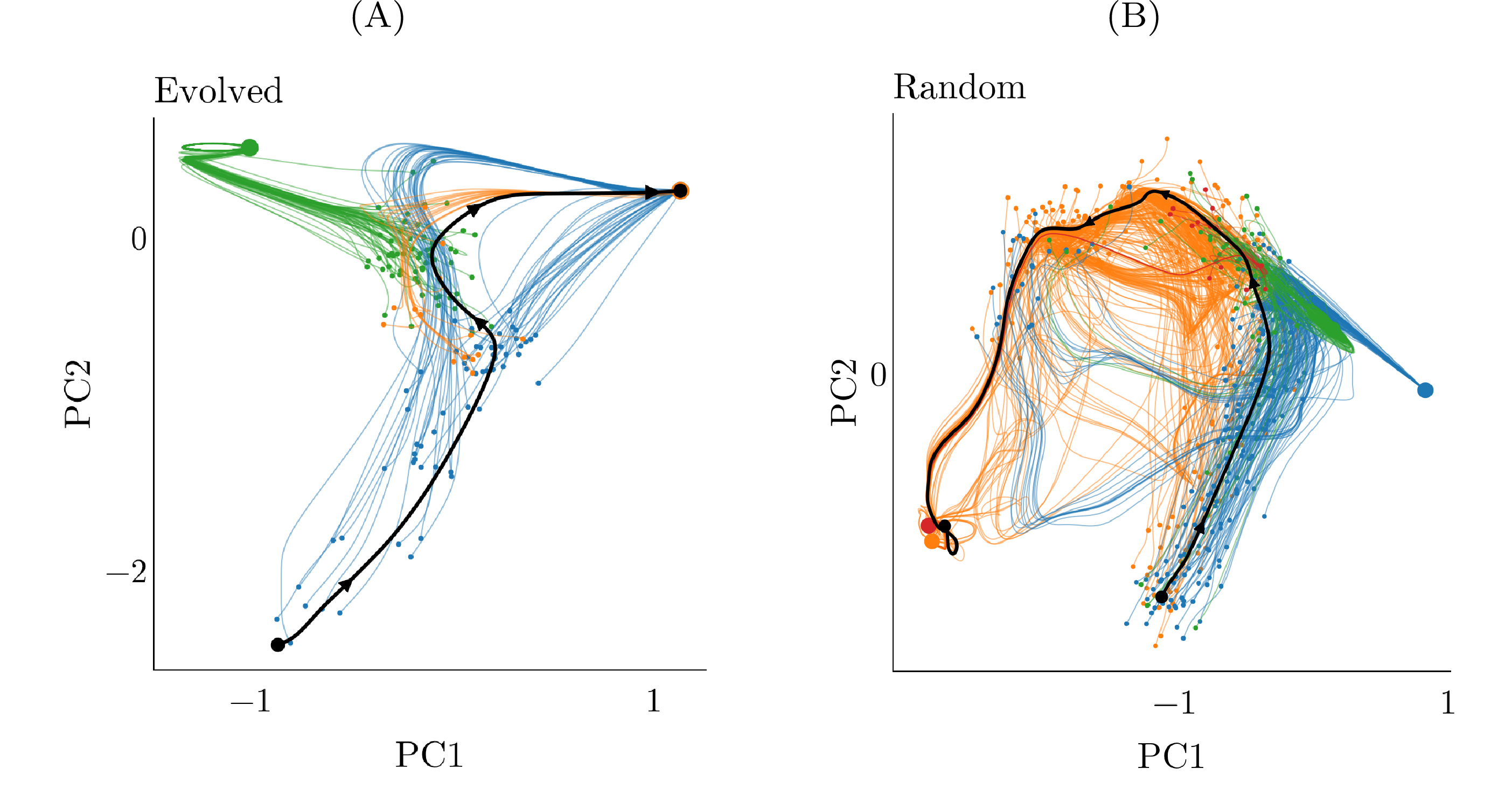

Supplement: S7 Fig — Different colors indicate trajectories that reach different attractors. Trajectories are analyzed by hierarchical clustering according to pairwise distance, and trajectories in the same cluster are shown in the same color. (TIF) [file pcbi.1014385.s008.tif]

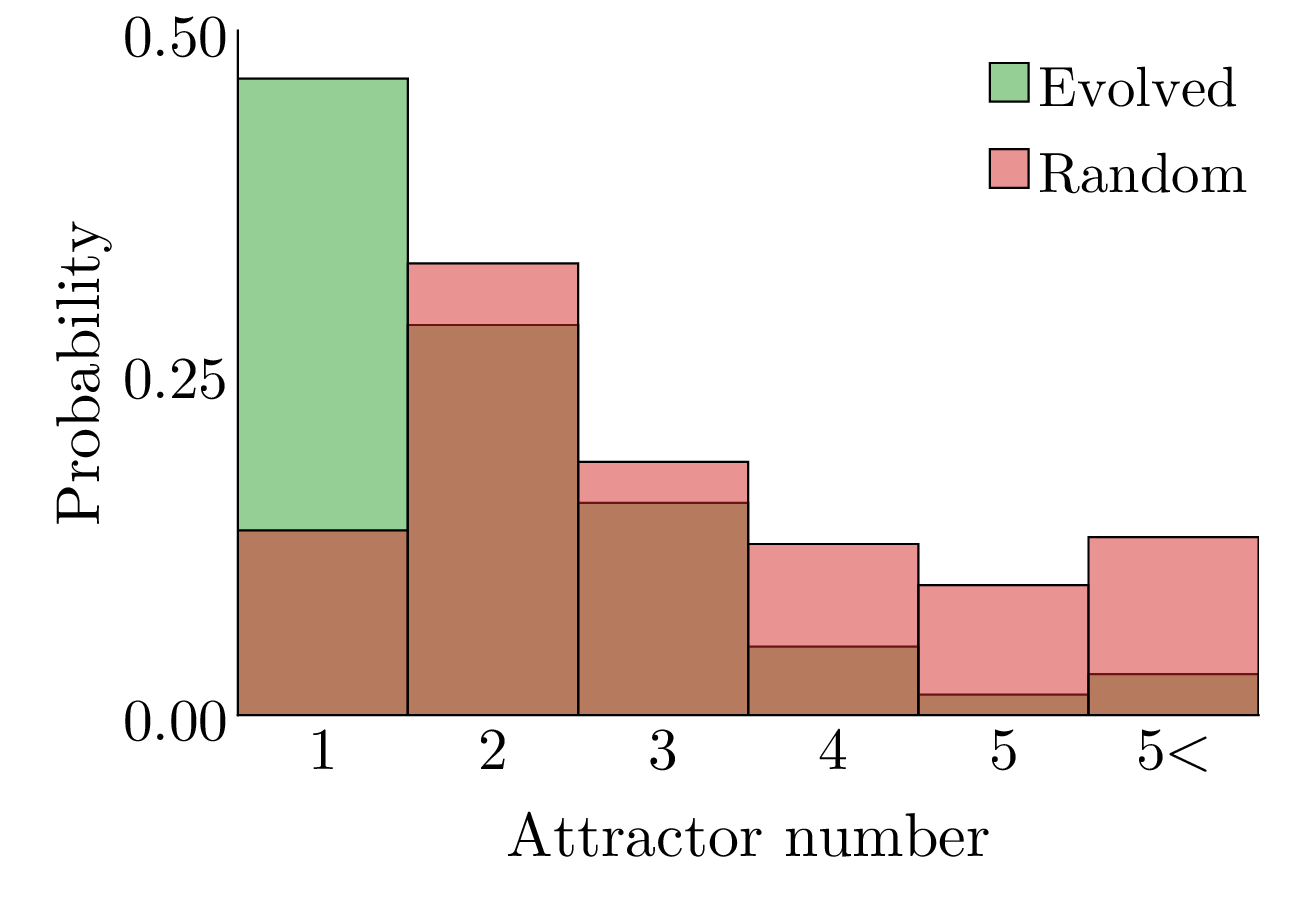

Supplement: S8 Fig — The fraction of individuals with more than five attractors is represented as a single bar labeled 5 < . (TIF) [file pcbi.1014385.s009.tif]

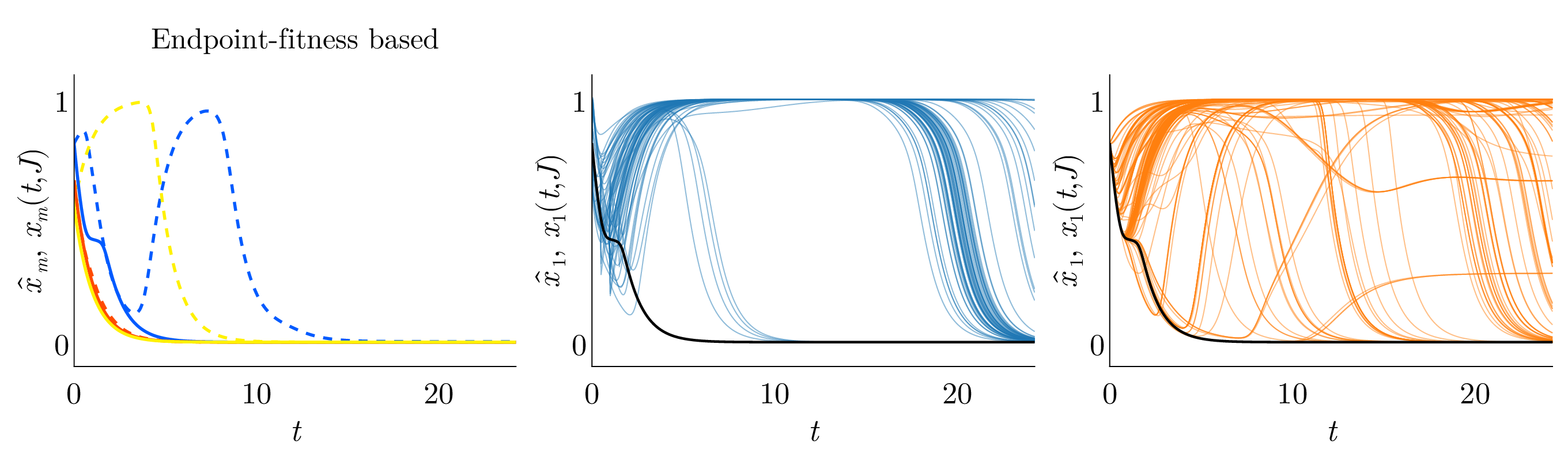

Supplement: S9 Fig — (TIF) [file pcbi.1014385.s010.tif]
