# Supplementary material for: Evolution of phenocopying in a dynamical model of developmental trajectories
Source: PLoS Comput Biol. 2026 Jun 9;22(6):e1014385. doi: 10.1371/journal.pcbi.1014385 (PMC13262954; doi:10.1371/journal.pcbi.1014385)
Supplement: Attachment — Submitted filename: Response to Reviewers.pdf [file pcbi.1014385.s014.pdf]

Additional Editor Comments:

All reviewers found this topic interesting, but they raised several critical concerns regarding this manuscript. For example, one of our reviewers asked authors to clarify how the values of involved parameters were selected, and some of reviewers agreed that authors should conduct more rigourous experiments to evluate the performance of their model. Moreover, more implementation details are suggested to be inlcuded for readers to better understand the novelty of this work.

We thank the editor and referees for their detailed comments and reading of the manuscript. There were many common themes in the referees' criticism and we have attempted to address them in our resubmission. The list of major changes we have made are as follows:

1) We now justify our parameter choices better. We also report results in the SI for additional parameter values.
2) We have added a new Figure (Figure 6 in the new manuscript) that more directly connects the results of our model with experiments.
3) We have addressed the potential limitation of comparing distributions in a lower dimensional space in two ways. First, by increasing the number of dimensions kept and verifying that there are no noticeable differences in the results and second by comparing our results with an alternative metric that directly utilizes the distance matrix in the full space.
4) We have tested our model in several ways e.g. by adding noise and by changing the definition of fitness and reported the results. We have also added other statistical tests e.g. sparsity statistics of the matrix J and attractor number.
5) Finally, we have edited the manuscript including figures/captions for clarity and added a new paragraph in the introduction more directly referencing recent literature and placing our work in context.

We give a more detailed reply below.

Reviewer #1: This paper presents a computational model to study the phenomenon of phenocopying, where environmental perturbations produce phenotypic changes that mimic genetic mutations. The authors develop an evolutionary model that evolves developmental trajectories to follow a specific reference trajectory, and they explore the effects of internal and external perturbations on these trajectories. The results demonstrate that evolution can lead to robustness against both types of perturbations, and phenocopying emerges as an unintended but significant outcome, providing insights into the dynamics of developmental systems and their potential applications in understanding genetic and environmental interactions.
Overall, the study is generally conceived and the manuscript is hazily written, and several crucial concerns/issues should be touched.

1. Overall, the grammar is mostly clear, but there are some areas that could benefit from improved sentence structure. For example, in the sentence "In Drosophila, they produced changes in bristles, wings, eyes etc.," it could be clearer to specify "such as" rather than "etc." for better readability.

We appreciate the reviewer's careful reading of our manuscript. We have replaced etc. with such as and made several other changes to the manuscript to improve readability.

2. The derivation of the formulas in the Methods section, particularly the ordinary differential equations (ODEs), is clear in terms of structure. However, the rationale behind the use of specific parameters, like "$\beta = 40$" in equation (1), is not explained in detail. Providing more context for why this value was chosen would improve the clarity and rigor of the explanation. Also, the logic of how the fitness function is calculated and evolved is presented clearly but could benefit from a more detailed explanation on how perturbations interact with the system in the simulation.

We have added a sentence to explain why we set beta = 40. We have also added other sentences to explain the parameter choices and conditions under which the main conclusions of our manuscript can be reproduced.

3. If the study were to incorporate binary classification tasks, it would be crucial to use cross-validation methods such as k-fold cross-validation or leave-one-out cross-validation to assess model generalization. The paper does not mention any cross-validation or evaluation methods, which are essential for validation in computational modeling studies.

We appreciate the referee's point which we interpret to mean that we should test the validity of the model and assess how general the conclusions are. We have made several additions to the paper to address this. We now evaluate the robustness of our conclusions to a) changes in parameter values, b) changes to our definition of the fitness and c) addition of developmental noise. Our main results are robust to changes in parameter values and addition of small developmental noise but rely on defining the fitness over trajectories as we now show in the paper.

4. The experimental part of the study includes simulations of perturbations (both external and internal), which seem valid in terms of modeling and providing insight into phenocopying and robustness. However, there is no comparison of these results with real-world experimental data. Including comparisons with biological systems, such as Drosophila, would help prove the practicality of the model. The model needs to demonstrate how these simulated results relate to empirical studies to ensure its relevance.

There is a paucity of quantitative experimental studies on phenocopies and we hope to encourage more such studies through this paper. One of the main results of prior experimental work is that phenocopies depend strongly on the timing of perturbation. We now add a new figure to the paper (Figure 6) which demonstrates that this also holds for our model. However, we caution that our model is abstract and not directly meant to model specific phenocopies observed in a particular organism.

5. The manuscript does not reference any very recent models (2024/2025) in its comparison section. Including comparisons with newer studies in the same area would strengthen the case for the originality and advancement of the proposed model. For example, citing recent work in gene regulatory dynamics or perturbation modeling would better contextualize the novelty of this study.

We thank the referee for this suggestion and have added a new paragraph to the introduction citing more recent studies and placing our paper in that context.

6. Some terms like "J", "xi(t; J)", and "xm(t; J)" are used throughout the paper without detailed explanation, especially for readers unfamiliar with dynamical systems. A clearer definition of the variables and terms in both the model and results sections would reduce ambiguity. For instance, the role of "J" as a matrix and how it evolves across generations could be elaborated further.

We have now explained the matrix J more clearly. In the figures, we have removed references to these symbols to avoid confusion and/or explained them in the caption.

7. To improve this work, the authors could cite recent advancements in computational biology and system modeling, such as those in "10.1016/j.csbj.2024.06.032" and "10.1093/bib/bbac384", to enhance the theoretical framework and validation of their models. Additionally, referencing "10.1109/TCBBIO.2025.3610881" and "10.1002/advs.202512453" could provide insights into the latest methods in perturbation modeling and dynamical systems, further strengthening the model's robustness and practical relevance in real-world applications.

We thank the referee for this suggestion but could not understand the direct relevance of these papers to our study. Nevertheless, we have now cited more recent advancements that are relevant to the paper as per the referee's suggestions.

Reviewer #2: Uploaded as an attachment

1. Topic & Significance
Evaluation: Excellent
The topic is highly relevant and original, addressing a long-standing concept (phenocopies) that has been historically described experimentally but rarely treated theoretically. The focus on trajectory-level evolution rather than endpoint phenotypes is a major conceptual strength.
Strengths
Strong alignment with evo-devo theory and dynamical systems biology
Clear relevance to robustness, canalization, and genetic assimilation
Suitable for a high-impact computational biology journal

2. Introduction
Evaluation: Good, but needs sharpening
The introduction provides a solid historical and theoretical background, covering canalization, robustness, and classical phenocopy literature (Waddington, Goldschmidt). The motivation for a dynamical systems approach is appropriate.
Strengths
Well-referenced and scholarly
Logical progression from canalization → phenocopies → modeling gap
Limitations
The specific gap is stated implicitly rather than explicitly
The novelty relative to recent theoretical models could be emphasized more clearly
The introduction could better articulate why trajectory-level evolution is essential, rather than optional
Recommendation
Add a clear "knowledge gap" paragraph at the end of the introduction
Explicitly state how this model goes beyond prior robustness or canalization models

We have added a paragraph in the introduction referencing more recent studies and clearly articulating how we go beyond prior models which do not evolve trajectories to address this concern.

3. Research Question
Evaluation: Conceptually strong, but not explicitly framed
The study addresses the following implicit research questions:
Can phenocopying emerge as an evolutionary property without direct selection?
Do internal (genetic) and external (environmental) perturbations converge in their effects after evolution?
Does evolution simplify basin structure in high-dimensional developmental systems?
Issue
These questions are not explicitly stated as formal research questions or hypotheses.

Recommendation
Add 1–2 explicit research questions or hypotheses at the end of the Introduction to improve clarity and reviewer accessibility.

Following the referee's suggestion, we have added explicit questions that we are addressing in the manuscript in the introduction.

4. Identified Gap
Evaluation: Strong and valid
The manuscript convincingly identifies a theoretical gap:
Phenocopying is well documented experimentally but lacks a general theoretical framework.
This gap is legitimate and well justified.
Strength
Clear contrast between extensive experimental literature and limited theoretical modeling
Proper justification for computational abstraction
Minor Suggestion
Briefly acknowledge limitations of abstraction (e.g., absence of molecular noise or explicit gene regulation)

We have added a sentence in the discussion mentioning that our model is abstract but these ideas could be tested for more explicit models of gene regulation.

5. Methodology
Evaluation: Strong but requires clarification in places
The dynamical systems model and evolutionary framework are technically sound and appropriate.
Strengths
Clear separation of development (ODE dynamics) and evolution (parameter selection)
Use of trajectory-based fitness is innovative
Inclusion of null model (random sampling via McMC) is excellent
Perturbation framework (internal vs external) is well designed
Concerns
Parameter justification
Choice of $N=40$, $M=3$, $\beta=40$, mutation rate, and perturbation magnitude $\Delta=0.25$ lack justification or sensitivity analysis.
No stochasticity
Absence of noise in development is a strong assumption and should be discussed.
Generality
Only a limited set of reference trajectories is explored.
Recommendation
Add a short sensitivity analysis or justification paragraph
Explicitly discuss how results might change with noise or alternative parameter choices

We have added several new results to the paper to address these concerns. We now try varying the population size and the mutation rate and report the results in the SI (Figure S6). We also try adding developmental noise and show the results in the SI (Figure S4). We have added justification for the parameter values chosen on introducing the model. We find that these additions do not change the main conclusions of our work. However, we also add a paragraph to mention that more work will be needed to explore how these parameters affect detailed properties of phenocopies which is beyond the scope of this work.

6. Results
Evaluation: Very strong
The results are clearly presented, logically ordered, and convincingly support the claims.
Strengths
Robust comparison between evolved and randomly sampled individuals
Clear demonstration of:
Increased robustness
Emergence of alternative trajectories
Overlap between internal and external perturbations
Effective use of MDS and basin analysis
Minor Issues
Some figures (e.g., Fig. 5–6) are information-dense and may challenge non-experts
Statistical significance testing could be made more explicit

We have changed the labels in the figures and no longer use mathematical equations to improve readability. We have also modified the captions to be clearer.

7. Discussion
Evaluation: Strong but could be more balanced
The discussion provides a convincing mechanistic interpretation based on basin simplification and low-dimensional structure.
Strengths

Clear link to canalization, genetic assimilation, and homeorhesis
Good integration with classical and modern literature
Limitations
Limited discussion of model assumptions
Experimental implications are mentioned but not developed
Recommendation
Add a short subsection: “Limitations and future directions”
Suggest concrete experimental tests (e.g., timing-dependent perturbations in model organisms)

We now have a new figure (Figure 6) in the paper more directly comparing our result to prior experiments (comparing the timing of perturbations, for example). While we see some

qualitative similarity with prior experimental results, we caution against direct quantitative comparison since our model is abstract and not built to be directly compared to experiments in a given organism. Nevertheless, the ideas could be applied for more realistic gene-regulatory networks as we now mention in the discussion.

8. Conclusion
Evaluation: Appropriate but brief
The conclusion correctly summarizes the main findings but could be strengthened.
Suggestion
Emphasize why this framework matters beyond phenocopies, e.g. for robustness, plasticity, or disease modeling.

We thank the referee for this comment and are gratified that they believe the framework could be extended to other areas like plasticity or disease modeling. However, we wish to be a bit more cautious in the interpretation of our work and therefore do not explicitly say this.

9. Language & Presentation
Evaluation: Very good
Language is clear, professional, and suitable for a Q1 journal
Minor typographical issues only
Mathematical notation is clear and consistent
Recommendation
Minor language polishing only (copy-editing level)

We have now made changes to the language throughout the paper to polish it as per the referee's suggestion.

Final Recommendation
Decision: Major Revision
Reasons for Major Revision
Research questions not explicitly stated
Methodological assumptions insufficiently justified
Limited discussion of limitations and generalizability
Summary of Required Revisions
Explicitly state research questions / hypotheses
Justify key model parameters or add sensitivity discussion
Expand discussion on limitations and experimental relevance

Minor improvements to figure clarity and statistical reporting

Reviewer #3: I appreciate the authors' effort to connect evolutionary dynamics in a gene regulatory network model with the emergence of phenocopy-like responses under perturbation. The topic is interesting and potentially impactful, but several aspects of the modeling, analysis, and reporting need clarification or additional validation before I can assess the strength of the main claims.

I am concerned there may be a sign or definition inconsistency in the evolutionary selection scheme, because fitness is defined as negative trajectory error so better individuals should correspond to less negative values, yet the reported selection probability form appears to assign higher selection probability to more negative fitness values unless there is an unreported convention or a sign error in the manuscript.

We thank the referee for catching this and indeed had a sign error in the manuscript which we have now corrected.

I do not think the comparison between evolved trajectories and the multicanonical Monte Carlo "random functional" null model is currently controlled tightly enough, because it is unclear whether the two sets are matched on trajectory error to the reference, weight or sparsity statistics of the networks, stability characteristics, or effects of the random initialization of non-trajectory nodes, and this makes it difficult to attribute the reported differences specifically to evolutionary bias rather than distributional mismatch.

It is difficult for us to directly address the referee's concern because some level of distributional mismatch is inevitable due to the differences in evolutionary and random sampling. However, we decided to additionally test whether the functional individuals obtained through both methods are truly close to the trajectory and explicitly state the criteria for the level of proximity considered acceptable. We have therefore added an additional figure in the SI (Figure S1) and a paragraph addressing these points. We also mention that random initialization does not contribute as we do not vary the initial conditions once they are set. We have also tested for the sparsity statistics of the networks as per the referee's suggestion and do not find a major difference in the two sampling methods (Figure S2).

I find the perturbation protocols underspecified in ways that affect interpretation, because it is not clearly stated whether external perturbations target one node or multiple nodes, how the perturbed node and time are selected, how state bounds are handled after applying the perturbation, and whether perturbations apply to trajectory nodes versus hidden nodes, and the internal perturbation procedure appears to rely on filtering for extreme outcomes rather than defining perturbation magnitude directly.

For the external perturbation, we target all the nodes and do not set boundaries for simplicity. As was mentioned in the main text, we conducted perturbation experiments at each of the 50 equally spaced time points obtained by dividing the trajectory. Since the perturbations are in a high dimensional space, perturbations of equal magnitudes can have very different effects along different parts of the trajectory. It is therefore necessary for us to filter extreme outcomes rather

than to define a consistent “magnitude” (see also our reply below). We have added text to detail the protocol for external perturbations.

I am not fully convinced by the operational definition of phenocopying as currently quantified, because the main statistics rely on an MDS embedding and density measures in the embedded space, and key implementation details for estimating densities are missing while the conclusions could be sensitive to the trajectory distance definition, embedding method, truncation to a fixed number of modes, and smoothing or binning choices, and overlap in an embedding does not necessarily imply strong similarity in the original high-dimensional trajectory space.

We appreciate the referee’s concern about evaluating the results in a low-dimensional space. We add two new results to the paper to address this concern. First, we evaluate our results by increasing the number of MDS dimensions kept and find that they are consistent. Second, we evaluate phenocopying more directly using the distance matrix itself and a different metric. We show these results in the SI (Figure S7). We now discuss this in the results.

I am concerned that the treatment of large perturbations is outcome-conditioned, because selecting only trajectories that deviate sufficiently far can bias the apparent structure and overlap and can also make evolved versus random comparisons unfair if tail behavior differs, so I would prefer “large” to be defined by intervention magnitude rather than by post hoc filtering on the response.

While we appreciate the referee’s concern, a perturbation can not be considered “large” in our model simply from the magnitude. This is because the definition of “large” can vary along the trajectory and along different directions. We have therefore added a paragraph to explain the reason we adopt post-hoc filtering. This is biologically relevant since large in that context is also operationally defined as a perturbation that leads to an observed phenotypic change. We now also quantify the dependency of the phenocopies on the strength of external perturbations in a new figure (Figure 6). We agree that this makes it difficult to precisely test the statistics of such perturbations but, in our view, does not affect the main results that evolved and random systems have distinct behaviour and the dynamical systems viewpoint that we offer.

I view the absence of developmental noise as a limitation for claims related to canalization and robustness, because deterministic shocks alone do not capture robustness to stochastic variation, and it remains unclear whether the observed phenocopying patterns persist under modest dynamical noise or parameter noise during development.

We agree with the referee’s point and have now added an additional figure testing our results both for changes in parameters and a small amount of dynamical noise. We find phenocopying can still emerge under such situations and the results are presented in the SI (Figure S6). We have also added a line in the discussion that observes that further study is needed to properly characterize how parameter values can affect specific properties of the phenocopies.

I think the claim that evolution simplifies basin structure is currently supported mainly by low-dimensional projections and qualitative basin visualizations, which can be misleading for high-dimensional dynamical systems, and I would like to see quantitative support such as attractor counts, basin volume estimates from many initial conditions, or stability and sensitivity metrics.

It is not clear that basin volume estimates are directly relevant because we start from fixed initial conditions (corresponding to early stages of a developmental trajectory). We agree that attractor counts could be one of the criteria to verify our hypothesis. We therefore compare the attractor numbers for individuals obtained from evolutionary and random sampling and have added these results to the SI (Figure S9).

Reviewer #4: Manuscript Number: PCOMPBIOL-D-25-02495
Title: Evolution of phenocopying in a dynamical model of developmental trajectories

Thank you for the opportunity to review this manuscript. The paper presents a theoretical and computational study of phenocopying using an abstract dynamical systems model of developmental trajectories. By evolving regulatory networks to follow a target trajectory, the authors investigate how robustness, canalization, and phenocopying emerge as properties of evolved systems. The study combines evolutionary simulations, perturbation analyses, and dynamical systems interpretations to provide insight into a long-standing question in evolutionary developmental biology. Overall, the manuscript is clearly written, methodologically sound, and addresses an underexplored theoretical problem. However, several issues should be addressed before publication. Please find my comments below:

1. The study addresses phenocopying from a dynamical systems perspective, which is relatively underexplored and represents a meaningful theoretical contribution. However, the manuscript would benefit from a clearer positioning relative to prior computational work on canalization, robustness, and evolved gene regulatory networks. Explicitly stating how this work advances beyond earlier robustness-focused models would strengthen the contribution.

We now more explicitly say that prior work has not looked at evolving trajectories. Further, we also make our questions more explicit in the new introduction and add a new paragraph that places our work in the context of recent related work.

2. The model is intentionally abstract, which is appropriate for a theoretical study. Nonetheless, the authors should more clearly discuss how the results can be generalized to real developmental gene regulatory networks, including limitations arising from the lack of noise, fixed network size, and simplified fitness definition.

We have added lines in the discussion both on the simplified fitness as well as the abstract nature of the model.

3. While the evolutionary framework is well described, the manuscript would benefit from additional discussion or supplementary analyses addressing sensitivity to key parameters (e.g., population size, mutation rate, selection strength, trajectory dimensionality). This would help demonstrate that the reported phenomena are robust rather than parameter-specific.

We have added new results which test our conclusions against changes in parameters including the population size and mutation rate. These are now reported in the SI (Figure S6). We have also added a line in the discussion that a more systematic study is required to fully address how the properties of phenocopies depend on these various parameters.

4. Phenocopying is convincingly demonstrated qualitatively and through MDS-based analyses. However, the manuscript could be strengthened by introducing a more explicit quantitative metric for phenocopy similarity, beyond overlap in reduced-dimensional space.

We now test our results with an alternative metric for phenocopies that directly uses the distance matrix. This is reported in the SI (Figure S7). However, we still retain the MDS visualization in the main text as it makes the phenomenon visually clear and easier to understand.

5. Although this is a theoretical paper, there is no ablation-style analysis examining the role of specific modeling choices (e.g., trajectory-based fitness vs endpoint fitness, internal vs external perturbation structure). Including such analyses would help isolate which ingredients are essential for the emergence of phenocopying.

We thank the referee for this suggestion. As previously written, we have now tested the model against changes in evolutionary parameters. Further, we now test to see if an endpoint based fitness is sufficient to evolve phenocopies. We find that, in our framework, such fitness produces very simple trajectories. While phenocopies may still be observed in principle, they are much less frequent than in our trajectory based fitness. We also now mention in the discussion that more work is needed to understand how the fitness criteria will affect our results.

6. Several figures are information-dense and require careful reading. Improving label sizes, clarifying legends, and explicitly stating what should be inferred from each panel would enhance accessibility, particularly for readers unfamiliar with dynamical systems theory.

We have now changed all the figure labels and increased their sizes. We have also changed the captions to remove mathematical symbols and improve readability. We have also modified certain figures to make them more accessible.

7. The current data availability statement does not meet PLOS Computational Biology requirements. Data cannot be “available upon request.” The authors must provide unrestricted access by depositing all processed datasets, including occurrence points, incidence series, environmental layers, and extraction scripts, as well as all model code, in a public repository with a permanent DOI, such as Zenodo.

We have uploaded and published our code in Zenodo with DOI (https://doi.org/10.5281/zenodo.18824124).

8. Some sections of the Results and Discussion are quite dense. Light editing for conciseness would improve readability without reducing technical depth.

We have made small edits to the entire manuscript and are happy to make further edits to specific sections if they remain unclear.

9. While generally readable, the manuscript would benefit from careful language editing. Some sentences are awkwardly constructed, and clarifying the narrative flow would improve overall readability.

We have made small edits to the language in the entire manuscript to improve readability.
