# Supplementary material for: Evolution of phenocopying in a dynamical model of developmental trajectories
Source: PLoS Comput Biol. 2026 Jun 9;22(6):e1014385. doi: 10.1371/journal.pcbi.1014385 (PMC13262954; doi:10.1371/journal.pcbi.1014385)
Supplement: Attachment — Submitted filename: Referee Response 2nd Revision.pdf [file pcbi.1014385.s015.pdf]

Reviewer #1: We suggest that the authors provide clear indications of the changes made and their sources in the response to reviewers. For example, some references suggested in #issue7 are difficult to trace in the revised manuscript. Clearly linking each revision to its corresponding location and citation will greatly facilitate the reviewers' further evaluation.

With regard to Issue #7, we had added a new paragraph to the Introduction (Line 65) and References 29, 30, 31. We had also added Ref. 49 in the discussion.

We also here briefly note the changes made with regard to the concerns raised by the referee in the previous report. Note that line numbers refer to the newly revised version. Detailed answers were provided in the previous reply.

#1 Several changes were made including Line 47 on page 1
#2 Line 92, and paragraph starting from Line 104
#3 Fig. S6, S9, S4
#4 Fig. 7 + Line 291 in Discussion
#5 Ref. 29, 30, 31, 49
#6 Line 93
#7 We could not directly understand the relevance of the papers the referee mentioned to our study. We have nevertheless cited Refs. 29, 30, 31 and 49 as recent studies related to this topic. We also put a new paragraph in the introduction (Line 65)

Reviewer #3: The revised manuscript is improved and the authors have made a clear effort to address several of the main concerns raised in the first review. In particular, the added clarifications and supplementary analyses strengthen the presentation, but some important issues remain unresolved and a few conclusions still appear stronger than the current evidence supports.

1. The null-model comparison is still not controlled tightly enough. The revision adds checks for fitness proximity and sparsity, but the central concern remains because the random functional networks are still not matched to the evolved networks on broader dynamical properties before perturbation. Since the main claim depends on attributing the difference to evolution rather than to residual distributional mismatch between the two ensembles, this part still needs a more controlled comparison or a stronger limitation statement.

We understand the referee's concerns. We have added an additional figure where we look at the fraction of each value -1, 0, 1 in the network (Fig. S2). We have now added an explicit limitation statement that we do not control for all dynamical properties between the two sampling methods (Line 138). Our view is that some level of distributional mismatch of dynamical properties between the two ensembles is inevitable and even informative. For example, the effect of small perturbations differs between the two ensembles as we later show and hence their stability characteristics differ. This is why we prefer only to test for elementary properties and do not match dynamical properties.

2. The treatment of large perturbations is still outcome-conditioned, and this remains a major interpretive weakness. The manuscript explicitly keeps only trajectories that are sufficiently far from the base trajectory after perturbation, then analyzes overlap and phenocopy structure on that filtered set. That makes the phenocopy statistics partly conditional on the response itself, which can inflate apparent structure and makes the evolved versus random comparison harder to interpret cleanly. The added explanation helps, but it does not remove the bias introduced by post hoc selection.

We appreciate the referee's concern about outcome conditioning. However, we are only able to define "phenocopies" after conditioning on the outcome. As stated previously, we have no clear way of defining a perturbation to be "large" except by looking at the outcome. We have added a line in the discussion (Line 265) that it would be interesting to study if such a correspondence could be established to find the kinds of perturbations that lead to large distances from the base trajectory (phenocopies).

For now, we have rephrased this paragraph and made it very explicit that we are studying the differences in the two ensembles conditioned on the outcome. This is necessary because we are interested in studying the statistics of phenocopies (and not of large perturbations, which may or may not produce phenocopies). We have also added a new figure showing the cumulative distribution function of the distance from the base trajectory in the two ensembles, as in Fig. 4D and 4E. It is clear that there are two differences between the two ensembles, the

probability of having a distance greater than a threshold is different as is the structure conditional on the distance being greater than a threshold. We now state this explicitly in the manuscript and also add a line in the discussion that our results are conditioned on the distance being greater than a threshold (Line 169).

3. The phenocopy metric is improved but still not fully convincing as a primary quantitative basis for the strongest claims. The authors now add higher-dimensional MDS checks and a distance-matrix-based alternative, which is a useful improvement. Even so, the main text still leans heavily on overlap and localization summaries whose biological interpretation remains indirect. The paper would be stronger if it defined phenocopying in a more direct trajectory-level way and made that the main result rather than a supplementary robustness check.

We follow the referee's suggestion and include our trajectory level metric in the main text. The MDS is now retained for visual purposes only.

4. The claim that evolution simplifies basin structure is still stronger than the quantitative evidence currently supports. The new attractor-count comparison is helpful, but attractor number alone is not enough to establish basin simplification in a high-dimensional system. The manuscript still relies mainly on projected phase portraits and qualitative basin arguments for this mechanistic conclusion. I would ask the authors to soften this claim or add more direct quantitative basin-level evidence.

We appreciate the referee's concern and have softened our claims in several parts. We have removed reference to "simplification" in the abstract and author summary. We have added a statement that the projected space does not capture the full basin complexity (Line 261). We have also softened the claim in the discussion (Line 339).

A full exploration would require a broader framework in nonlinear dynamics which is beyond the scope of the present study.

5. The perturbation design is clearer now, but it also reveals a new conceptual concern. External perturbations are applied to all nodes at once, with no state boundaries, and at many times along the trajectory. That is mathematically permissible, but it makes the environmental perturbation model extremely broad and not especially comparable to localized or structured developmental insults. Because the biological interpretation of phenocopying is one of the paper's motivations, the manuscript should discuss more explicitly how this global perturbation protocol may shape the observed overlap with internal perturbations.

The referee's point is clearly a limitation of our modeling for perturbations. We now add a discussion of this point (Line 265) mentioning it as a limitation. Our aim here is to introduce the simplest perturbation scheme where the phenomena can be explored. One interesting point

about the biological literature is that these perturbations were just generic stresses (like heat shocks, or ether treatment). Indeed, what kinds of perturbations lead to phenocopies is not only interesting from a biological point of view, but is also interesting for point #2 that the referee raises.

6. The robustness analyses are still somewhat narrow relative to the generality of the claims. The revision adds sensitivity to population size, mutation rate, noise, and endpoint fitness, which is valuable. But the main conclusions are still presented broadly while the explored reference trajectories, perturbation structures, and network settings remain limited. I would ask the authors either to narrow the wording of the main claims or to frame the conclusions more explicitly as applying to this model class rather than to developmental systems in general.

We have added an explicit reference to the model and edited the abstract, author summary and Introduction to avoid giving this impression.

7. A new issue is that some of the key validation material appears to have been moved to the supplement while the main paper still draws strong mechanistic conclusions. The revisions rely on supplementary figures for the distance-matrix metric, parameter robustness, attractor counts, and endpoint-fitness comparison. Those additions are important enough that at least one of them, especially the direct non-MDS phenocopy metric, should be promoted into the main text because it directly addresses a central review concern.

As in point #3, we have moved the direct phenocopy metric to the main text and our analysis in the main text is done using this metric. The MDS figure is now only retained in the main text for visual purposes as we believe it can help give the reader intuition for the results. We have changed Fig. 5 and 6 accordingly.
