# Supplementary material for: Evolution of phenocopying in a dynamical model of developmental trajectories
Source: PLoS Comput Biol. 2026 Jun 9;22(6):e1014385. doi: 10.1371/journal.pcbi.1014385 (PMC13262954; doi:10.1371/journal.pcbi.1014385)
Supplement: Attachment — Submitted filename: Reviewer Response PLOS Comp Bio Resubmission 3.pdf [file pcbi.1014385.s016.pdf]

Reviewer #1: 1. The study relies entirely on computational simulations of developmental trajectories to investigate phenocopying. However, there is no experimental or observational data used to validate the model predictions, which makes it difficult to assess the biological relevance of the results. For high-impact publication, some form of empirical benchmarking or comparison to experimental phenocopy data is recommended.

The referee had previously raised the question of experimental data in their first report (Point #4 in their first report) to which we had responded by adding an additional figure to the paper (Figure 7) comparing our model to existing experimental data. Our aim here is not to study a specific developmental system, but rather to investigate theoretically whether a correspondence between environmental and genetic perturbations can generically emerge in evolved dynamical systems.

2. The gene regulatory network model uses idealized ordinary differential equations with uniform random perturbations. These assumptions ignore stochastic effects, epigenetic regulation, and intercellular interactions. The simplifications may limit the model's ability to capture realistic developmental dynamics, reducing confidence in the conclusions drawn.

We previously added an additional figure to test the model in the presence of stochasticity (Figure S6). Further, we follow previous work demonstrating that complex developmental systems can be modeled as trajectories (Ref. 26 in the paper), rather than modeling specific mechanisms which would vary across systems.

3. While the manuscript revisits classical concepts of canalization and robustness, the model largely reproduces known qualitative behaviors without providing new mechanistic insights or predictive power. Figures primarily illustrate trajectory overlaps without rigorous quantitative or biological interpretation, which diminishes the work's novelty and impact.

We are not simply revisiting classical concepts of canalization and robustness. The central question being studied in the manuscript is not robustness itself, but rather the structure of developmental trajectories when they are perturbed beyond the range to which they are robust by environmental or genetic perturbations. In the changes we made in the previous rounds, we added a new figure very quantitatively demonstrating the overlap between trajectories (Figure 6). We re-emphasize two novel conceptual results here: 1) Phenocopies can be obtained without being explicitly selected for, 2) The phenomenon is related to the basin structure (Fig. 8). This provides a dynamical systems mechanism for our observations.

Reviewer #3: The authors have addressed the major concerns raised in my previous reviews in a thorough and convincing manner, and these revisions are clearly incorporated throughout the final revised manuscript. Their responses are clear, well

organized, and adequately detailed, and the added clarifications and corrections have significantly improved the technical soundness, transparency, and overall consistency of the paper. I did not observe any remaining methodological concerns or major presentation issues that would undermine the validity of the findings or the credibility of the conclusions. At this stage, my recommendations are limited to minor editorial refinements, including careful proofreading for grammar, wording, and stylistic consistency, along with maintaining uniform terminology across the manuscript. Subject to these minor revisions, I consider the manuscript suitable for submission.

We thank the referee for their engagement with the manuscript. We have checked it carefully and made several changes to improve the grammar and wording.
