# Supplementary material for: Evolution of phenocopying in a dynamical model of developmental trajectories
Source: PLoS Comput Biol. 2026 Jun 9;22(6):e1014385. doi: 10.1371/journal.pcbi.1014385 (PMC13262954; doi:10.1371/journal.pcbi.1014385)
Supplement: Attachment — Submitted filename: TSR.docx [file pcbi.1014385.s011.docx]

***1. Topic & Significance***

**Evaluation:** Excellent

The topic is highly relevant and original, addressing a long-standing concept (phenocopies) that has been historically described experimentally but rarely treated theoretically. The focus on trajectory-level evolution rather than endpoint phenotypes is a major conceptual strength.

***Strengths***

1- Strong alignment with evo-devo theory and dynamical systems biology
2- Clear relevance to robustness, canalization, and genetic assimilation
3- Suitable for a high-impact computational biology journal

***2. Introduction***

**Evaluation:** Good, but needs sharpening

The introduction provides a solid historical and theoretical background, covering canalization, robustness, and classical phenocopy literature (Waddington, Goldschmidt). The motivation for a dynamical systems approach is appropriate.

***Strengths***

1- Well-referenced and scholarly
2- Logical progression from canalization → phenocopies → modeling gap

***Limitations***

1- The specific gap is stated implicitly rather than explicitly
2- The novelty relative to recent theoretical models could be emphasized more clearly
3- The introduction could better articulate why trajectory-level evolution is essential, rather than optional

**Recommendation**

1- Add a clear “knowledge gap” paragraph at the end of the introduction
2- Explicitly state how this model goes beyond prior robustness or canalization models

***3. Research Question***

**Evaluation:** Conceptually strong, but not explicitly framed

**The study addresses the following implicit research questions:**

1- Can phenocopying emerge as an evolutionary property without direct selection?
2- Do internal (genetic) and external (environmental) perturbations converge in their effects after evolution?
3- Does evolution simplify basin structure in high-dimensional developmental systems?

**Issue**

These questions are not explicitly stated as formal research questions or hypotheses.

**Recommendation**

Add 1–2 explicit research questions or hypotheses at the end of the Introduction to improve clarity and reviewer accessibility.

***4. Identified Gap***

**Evaluation:** Strong and valid

The manuscript convincingly identifies a **theoretical gap**:

Phenocopying is well documented experimentally but lacks a general theoretical framework.

This gap is legitimate and well justified.

***Strength***

Clear contrast between extensive experimental literature and limited theoretical modeling

Proper justification for computational abstraction

***Minor Suggestion***

Briefly acknowledge limitations of abstraction (e.g., absence of molecular noise or explicit gene regulation)

***5. Methodology***

**Evaluation:** Strong but requires clarification in places

The dynamical systems model and evolutionary framework are technically sound and appropriate.

***Strengths***

1- Clear separation of development (ODE dynamics) and evolution (parameter selection)
2- Use of trajectory-based fitness is innovative
3- Inclusion of null model (random sampling via McMC) is excellent
4- Perturbation framework (internal vs external) is well designed

***Concerns***

1. **Parameter justification**
   - Choice of N=40N = 40N=40, M=3M = 3M=3, β=40\beta = 40β=40, mutation rate, and perturbation magnitude Δ=0.25\Delta = 0.25Δ=0.25 lack justification or sensitivity analysis.
2. **No stochasticity**
   - Absence of noise in development is a strong assumption and should be discussed.
3. **Generality**
   - Only a limited set of reference trajectories is explored.

***Recommendation***

1- Add a short sensitivity analysis or justification paragraph
2- Explicitly discuss how results might change with noise or alternative parameter choices

***6. Results***

**Evaluation:** Very strong

The results are clearly presented, logically ordered, and convincingly support the claims.

**Strengths**

1- Robust comparison between evolved and randomly sampled individuals
2- Clear demonstration of:
   - Increased robustness
   - Emergence of alternative trajectories
   - Overlap between internal and external perturbations
3- Effective use of MDS and basin analysis

**Minor Issues**

- Some figures (e.g., Fig. 5–6) are information-dense and may challenge non-experts
- Statistical significance testing could be made more explicit

***7. Discussion***

**Evaluation:** Strong but could be more balanced

The discussion provides a convincing mechanistic interpretation based on basin simplification and low-dimensional structure.

***Strengths***

Clear link to canalization, genetic assimilation, and homeorhesis

Good integration with classical and modern literature

***Limitations***

Limited discussion of model assumptions

Experimental implications are mentioned but not developed

**Recommendation**

Add a short subsection: “Limitations and future directions”

Suggest concrete experimental tests (e.g., timing-dependent perturbations in model organisms)

***8. Conclusion***

**Evaluation:** Appropriate but brief

The conclusion correctly summarizes the main findings but could be strengthened.

***Suggestion***

Emphasize why this framework matters beyond phenocopies, e.g. for robustness, plasticity, or disease modeling.

**9. Language & Presentation**

**Evaluation:** Very good

Language is clear, professional, and suitable for a Q1 journal

Minor typographical issues only

Mathematical notation is clear and consistent

**Recommendation**

Minor language polishing only (copy-editing level)

**Final Recommendation**

**Decision:** **Major Revision**

**Reasons for Major Revision**

Research questions not explicitly stated

Methodological assumptions insufficiently justified

Limited discussion of limitations and generalizability

***Summary of Required Revisions***

1. Explicitly state research questions / hypotheses
2. Justify key model parameters or add sensitivity discussion
3. Expand discussion on limitations and experimental relevance
4. Minor improvements to figure clarity and statistical reporting
