# Supplementary material for: Evolution of phenocopying in a dynamical model of developmental trajectories
Source: PLoS Comput Biol. 2026 Jun 9;22(6):e1014385. doi: 10.1371/journal.pcbi.1014385 (PMC13262954; doi:10.1371/journal.pcbi.1014385)
Supplement: Attachment — Submitted filename: Review_PCOMPBIOL-D-25-02495.docx [file pcbi.1014385.s012.docx]

**Manuscript Number: PCOMPBIOL-D-25-02495**

**Title: Evolution of phenocopying in a dynamical model of developmental trajectories**

Thank you for the opportunity to review this manuscript. The paper presents a theoretical and computational study of phenocopying using an abstract dynamical systems model of developmental trajectories. By evolving regulatory networks to follow a target trajectory, the authors investigate how robustness, canalization, and phenocopying emerge as properties of evolved systems. The study combines evolutionary simulations, perturbation analyses, and dynamical systems interpretations to provide insight into a long-standing question in evolutionary developmental biology. Overall, the manuscript is clearly written, methodologically sound, and addresses an underexplored theoretical problem. However, several issues should be addressed before publication. Please find my comments below:

**1.** The study addresses phenocopying from a dynamical systems perspective, which is relatively underexplored and represents a meaningful theoretical contribution. However, the manuscript would benefit from a clearer positioning relative to prior computational work on canalization, robustness, and evolved gene regulatory networks. Explicitly stating how this work advances beyond earlier robustness-focused models would strengthen the contribution.

**2.** The model is intentionally abstract, which is appropriate for a theoretical study. Nonetheless, the authors should more clearly discuss how the results can be generalized to real developmental gene regulatory networks, including limitations arising from the lack of noise, fixed network size, and simplified fitness definition.

**3.** While the evolutionary framework is well described, the manuscript would benefit from additional discussion or supplementary analyses addressing sensitivity to key parameters (e.g., population size, mutation rate, selection strength, trajectory dimensionality). This would help demonstrate that the reported phenomena are robust rather than parameter-specific.

**4.** Phenocopying is convincingly demonstrated qualitatively and through MDS-based analyses. However, the manuscript could be strengthened by introducing a more explicit quantitative metric for phenocopy similarity, beyond overlap in reduced-dimensional space.

**5.** Although this is a theoretical paper, there is no ablation-style analysis examining the role of specific modeling choices (e.g., trajectory-based fitness vs endpoint fitness, internal vs external perturbation structure). Including such analyses would help isolate which ingredients are essential for the emergence of phenocopying.

**6.** Several figures are information-dense and require careful reading. Improving label sizes, clarifying legends, and explicitly stating what should be inferred from each panel would enhance accessibility, particularly for readers unfamiliar with dynamical systems theory.

**7.** The current data availability statement does not meet PLOS Computational Biology requirements. Data cannot be “available upon request.” The authors must provide unrestricted access by depositing all processed datasets, including occurrence points, incidence series, environmental layers, and extraction scripts, as well as all model code, in a public repository with a permanent DOI, such as Zenodo.

**8.** Some sections of the Results and Discussion are quite dense. Light editing for conciseness would improve readability without reducing technical depth.

9. While generally readable, the manuscript would benefit from careful language editing. Some sentences are awkwardly constructed, and clarifying the narrative flow would improve overall readability.
