# Supplementary material for: Evolution of phenocopying in a dynamical model of developmental trajectories
Source: PLoS Comput Biol. 2026 Jun 9;22(6):e1014385. doi: 10.1371/journal.pcbi.1014385 (PMC13262954; doi:10.1371/journal.pcbi.1014385)
Supplement: Peer review history

# Peer review: Decision Letter 0

**DOI:** 10.1371/journal.pcbi.1014385.r001

**Authors:** Lun Hu

6 Jan 2026

PCOMPBIOL-D-25-02495

Evolution of phenocopying in a dynamical model of developmental trajectories

PLOS Computational Biology

Dear Dr. Raju,

Thank you for submitting your manuscript to PLOS Computational Biology. After careful consideration, we feel that it has merit but does not fully meet PLOS Computational Biology's publication criteria as it currently stands. Therefore, we invite you to submit a revised version of the manuscript that addresses the points raised during the review process.

Please submit your revised manuscript by Mar 08 2026 11:59PM. If you will need more time than this to complete your revisions, please reply to this message or contact the journal office at ploscompbiol@plos.org. When you're ready to submit your revision, log on to https://www.editorialmanager.com/pcompbiol/ and select the 'Submissions Needing Revision' folder to locate your manuscript file.

Please include the following items when submitting your revised manuscript:

* A letter that responds to each point raised by the editor and reviewer(s). You should upload this letter as a separate file labeled 'Response to Reviewers'. This file does not need to include responses to formatting updates and technical items listed in the 'Journal Requirements' section below.

* A marked-up copy of your manuscript that highlights changes made to the original version. You should upload this as a separate file labeled 'Revised Manuscript with Track Changes'.

* An unmarked version of your revised paper without tracked changes. You should upload this as a separate file labeled 'Manuscript'.

If you would like to make changes to your financial disclosure, competing interests statement, or data availability statement, please make these updates within the submission form at the time of resubmission. Guidelines for resubmitting your figure files are available below the reviewer comments at the end of this letter

We look forward to receiving your revised manuscript.

Kind regards,

Lun Hu

Academic Editor

PLOS Computational Biology

Natalia Komarova

Section Editor

PLOS Computational Biology

**Additional Editor Comments:**

All reviewers found this topic interesting, but they raised several critical concerns regarding this manuscript. For example, one of our reviewers asked authors to clarify how the values of involved parameters were selected, and some of reviewers agreed that authors should conduct more rigourous experiments to evluate the performance of their model. Moreover, more implementation details are suggested to be inlcuded for readers to better understand the novelty of this work.

**Journal Requirements:**

If the reviewer comments include a recommendation to cite specific previously published works, please review and evaluate these publications to determine whether they are relevant and should be cited. There is no requirement to cite these works unless the editor has indicated otherwise.

1) Please ensure that the CRediT author contributions listed for every co-author are completed accurately and in full.

At this stage, the following Authors/Authors require contributions: Yuuki Matsushita, and Archishman Raju. Please ensure that the full contributions of each author are acknowledged in the "Add/Edit/Remove Authors" section of our submission form.

The list of CRediT author contributions may be found here: https://journals.plos.org/ploscompbiol/s/authorship#loc-author-contributions

2) We ask that a manuscript source file is provided at Revision. Please upload your manuscript file as a .doc, .docx, .rtf or .tex. If you are providing a .tex file, please upload it under the item type u2018LaTeX Source Fileu2019 and leave your .pdf version as the item type u2018Manuscriptu2019.

3) Please upload all main figures as separate Figure files in .tif or .eps format. For more information about how to convert and format your figure files please see our guidelines:

https://journals.plos.org/ploscompbiol/s/figures

4) We have noticed that you have uploaded Supporting Information files, but you have not included a list of legends. Please add a full list of legends for your Supporting Information files after the references list.

5) Please amend your detailed Financial Disclosure statement. This is published with the article. It must therefore be completed in full sentences and contain the exact wording you wish to be published.

1) State the initials, alongside each funding source, of each author to receive each grant. For example: "This work was supported by the National Institutes of Health (####### to AM; ###### to CJ) and the National Science Foundation (###### to AM)."

2) State what role the funders took in the study. If the funders had no role in your study, please state: "The funders had no role in study design, data collection and analysis, decision to publish, or preparation of the manuscript."

3) If any authors received a salary from any of your funders, please state which authors and which funders..

If you did not receive any funding for this study, please simply state: u201cThe authors received no specific funding for this work.u201d

6) Kindly revise your competing statement in the online submission form to align with the journal's style guidelines: 'The authors declare that there are no competing interests.'

**Reviewers' comments:**

Reviewer's Responses to Questions

**Comments to the Authors:**

**Please note here if the review is uploaded as an attachment.**

Reviewer #1: This paper presents a computational model to study the phenomenon of phenocopying, where environmental perturbations produce phenotypic changes that mimic genetic mutations. The authors develop an evolutionary model that evolves developmental trajectories to follow a specific reference trajectory, and they explore the effects of internal and external perturbations on these trajectories. The results demonstrate that evolution can lead to robustness against both types of perturbations, and phenocopying emerges as an unintended but significant outcome, providing insights into the dynamics of developmental systems and their potential applications in understanding genetic and environmental interactions.

Overall, the study is general conceived and the manuscript is hazily written, and several crucial concerns/issues should be touched.

1. Overall, the grammar is mostly clear, but there are some areas that could benefit from improved sentence structure. For example, in the sentence "In Drosophila, they produced changes in bristles, wings, eyes etc.," it could be clearer to specify "such as" rather than "etc." for better readability.

2. The derivation of the formulas in the Methods section, particularly the ordinary differential equations (ODEs), is clear in terms of structure. However, the rationale behind the use of specific parameters, like "β = 40" in equation (1), is not explained in detail. Providing more context for why this value was chosen would improve the clarity and rigor of the explanation. Also, the logic of how the fitness function is calculated and evolved is presented clearly but could benefit from a more detailed explanation on how perturbations interact with the system in the simulation.

3. If the study were to incorporate binary classification tasks, it would be crucial to use cross-validation methods such as k-fold cross-validation or leave-one-out cross-validation to assess model generalization. The paper does not mention any cross-validation or evaluation methods, which are essential for validation in computational modeling studies.

4. The experimental part of the study includes simulations of perturbations (both external and internal), which seem valid in terms of modeling and providing insight into phenocopying and robustness. However, there is no comparison of these results with real-world experimental data. Including comparisons with biological systems, such as Drosophila, would help prove the practicality of the model. The model needs to demonstrate how these simulated results relate to empirical studies to ensure its relevance.

5. The manuscript does not reference any very recent models (2024/2025) in its comparison section. Including comparisons with newer studies in the same area would strengthen the case for the originality and advancement of the proposed model. For example, citing recent work in gene regulatory dynamics or perturbation modeling would better contextualize the novelty of this study.

6. Some terms like "J", "xi(t; J)", and "xm(t; J)" are used throughout the paper without detailed explanation, especially for readers unfamiliar with dynamical systems. A clearer definition of the variables and terms in both the model and results sections would reduce ambiguity. For instance, the role of "J" as a matrix and how it evolves across generations could be elaborated further.

7. To improve this work, the authors could cite recent advancements in computational biology and system modeling, such as those in "10.1016/j.csbj.2024.06.032" and "10.1093/bib/bbac384", to enhance the theoretical framework and validation of their models. Additionally, referencing "10.1109/TCBBIO.2025.3610881" and "10.1002/advs.202512453" could provide insights into the latest methods in perturbation modeling and dynamical systems, further strengthening the model’s robustness and practical relevance in real-world applications.

Reviewer #2: Uploaded as an attachment

Reviewer #3: I appreciate the authors’ effort to connect evolutionary dynamics in a gene regulatory network model with the emergence of phenocopy-like responses under perturbation. The topic is interesting and potentially impactful, but several aspects of the modeling, analysis, and reporting need clarification or additional validation before I can assess the strength of the main claims.

I am concerned there may be a sign or definition inconsistency in the evolutionary selection scheme, because fitness is defined as negative trajectory error so better individuals should correspond to less negative values, yet the reported selection probability form appears to assign higher selection probability to more negative fitness values unless there is an unreported convention or a sign error in the manuscript.

I do not think the comparison between evolved trajectories and the multicanonical Monte Carlo “random functional” null model is currently controlled tightly enough, because it is unclear whether the two sets are matched on trajectory error to the reference, weight or sparsity statistics of the networks, stability characteristics, or effects of the random initialization of non-trajectory nodes, and this makes it difficult to attribute the reported differences specifically to evolutionary bias rather than distributional mismatch.

I find the perturbation protocols underspecified in ways that affect interpretation, because it is not clearly stated whether external perturbations target one node or multiple nodes, how the perturbed node and time are selected, how state bounds are handled after applying the perturbation, and whether perturbations apply to trajectory nodes versus hidden nodes, and the internal perturbation procedure appears to rely on filtering for extreme outcomes rather than defining perturbation magnitude directly.

I am not fully convinced by the operational definition of phenocopying as currently quantified, because the main statistics rely on an MDS embedding and density measures in the embedded space, and key implementation details for estimating densities are missing while the conclusions could be sensitive to the trajectory distance definition, embedding method, truncation to a fixed number of modes, and smoothing or binning choices, and overlap in an embedding does not necessarily imply strong similarity in the original high-dimensional trajectory space.

I am concerned that the treatment of large perturbations is outcome-conditioned, because selecting only trajectories that deviate sufficiently far can bias the apparent structure and overlap and can also make evolved versus random comparisons unfair if tail behavior differs, so I would prefer “large” to be defined by intervention magnitude rather than by post hoc filtering on the response.

I view the absence of developmental noise as a limitation for claims related to canalization and robustness, because deterministic shocks alone do not capture robustness to stochastic variation, and it remains unclear whether the observed phenocopying patterns persist under modest dynamical noise or parameter noise during development.

I think the claim that evolution simplifies basin structure is currently supported mainly by low-dimensional projections and qualitative basin visualizations, which can be misleading for high-dimensional dynamical systems, and I would like to see quantitative support such as attractor counts, basin volume estimates from many initial conditions, or stability and sensitivity metrics.

Reviewer #4: Manuscript Number: PCOMPBIOL-D-25-02495

Title: Evolution of phenocopying in a dynamical model of developmental trajectories

Thank you for the opportunity to review this manuscript. The paper presents a theoretical and computational study of phenocopying using an abstract dynamical systems model of developmental trajectories. By evolving regulatory networks to follow a target trajectory, the authors investigate how robustness, canalization, and phenocopying emerge as properties of evolved systems. The study combines evolutionary simulations, perturbation analyses, and dynamical systems interpretations to provide insight into a long-standing question in evolutionary developmental biology. Overall, the manuscript is clearly written, methodologically sound, and addresses an underexplored theoretical problem. However, several issues should be addressed before publication. Please find my comments below:

1. The study addresses phenocopying from a dynamical systems perspective, which is relatively underexplored and represents a meaningful theoretical contribution. However, the manuscript would benefit from a clearer positioning relative to prior computational work on canalization, robustness, and evolved gene regulatory networks. Explicitly stating how this work advances beyond earlier robustness-focused models would strengthen the contribution.

2. The model is intentionally abstract, which is appropriate for a theoretical study. Nonetheless, the authors should more clearly discuss how the results can be generalized to real developmental gene regulatory networks, including limitations arising from the lack of noise, fixed network size, and simplified fitness definition.

3. While the evolutionary framework is well described, the manuscript would benefit from additional discussion or supplementary analyses addressing sensitivity to key parameters (e.g., population size, mutation rate, selection strength, trajectory dimensionality). This would help demonstrate that the reported phenomena are robust rather than parameter-specific.

4. Phenocopying is convincingly demonstrated qualitatively and through MDS-based analyses. However, the manuscript could be strengthened by introducing a more explicit quantitative metric for phenocopy similarity, beyond overlap in reduced-dimensional space.

5. Although this is a theoretical paper, there is no ablation-style analysis examining the role of specific modeling choices (e.g., trajectory-based fitness vs endpoint fitness, internal vs external perturbation structure). Including such analyses would help isolate which ingredients are essential for the emergence of phenocopying.

6. Several figures are information-dense and require careful reading. Improving label sizes, clarifying legends, and explicitly stating what should be inferred from each panel would enhance accessibility, particularly for readers unfamiliar with dynamical systems theory.

7. The current data availability statement does not meet PLOS Computational Biology requirements. Data cannot be “available upon request.” The authors must provide unrestricted access by depositing all processed datasets, including occurrence points, incidence series, environmental layers, and extraction scripts, as well as all model code, in a public repository with a permanent DOI, such as Zenodo.

8. Some sections of the Results and Discussion are quite dense. Light editing for conciseness would improve readability without reducing technical depth.

9. While generally readable, the manuscript would benefit from careful language editing. Some sentences are awkwardly constructed, and clarifying the narrative flow would improve overall readability.

**********

**Have the authors made all data and (if applicable) computational code underlying the findings in their manuscript fully available?**

The PLOS Data policy requires authors to make all data and code underlying the findings described in their manuscript fully available without restriction, with rare exception (please refer to the Data Availability Statement in the manuscript PDF file). The data and code should be provided as part of the manuscript or its supporting information, or deposited to a public repository. For example, in addition to summary statistics, the data points behind means, medians and variance measures should be available. If there are restrictions on publicly sharing data or code —e.g. participant privacy or use of data from a third party—those must be specified.

Reviewer #1: None

Reviewer #2: None

Reviewer #3: Yes

Reviewer #4: **No:**

**********

PLOS authors have the option to publish the peer review history of their article (what does this mean?). If published, this will include your full peer review and any attached files.

If you choose “no”, your identity will remain anonymous but your review may still be made public.

**Do you want your identity to be public for this peer review?**   For information about this choice, including consent withdrawal, please see our Privacy Policy.

Reviewer #1: No

Reviewer #2: No

Reviewer #3: No

Reviewer #4: No

[NOTE: If reviewer comments were submitted as an attachment file, they will be attached to this email and accessible via the submission site. Please log into your account, locate the manuscript record, and check for the action link "View Attachments". If this link does not appear, there are no attachment files.]

**Figure resubmission:**

While revising your submission, we strongly recommend that you use PLOS’s NAAS tool (https://ngplosjournals.pagemajik.ai/artanalysis) to test your figure files. NAAS can convert your figure files to the TIFF file type and meet basic requirements (such as print size, resolution), or provide you with a report on issues that do not meet our requirements and that NAAS cannot fix.

After uploading your figures to PLOS’s NAAS tool - https://ngplosjournals.pagemajik.ai/artanalysis, NAAS will process the files provided and display the results in the "Uploaded Files" section of the page as the processing is complete. If the uploaded figures meet our requirements (or NAAS is able to fix the files to meet our requirements), the figure will be marked as "fixed" above. If NAAS is unable to fix the files, a red "failed" label will appear above. When NAAS has confirmed that the figure files meet our requirements, please download the file via the download option, and include these NAAS processed figure files when submitting your revised manuscript.

**Reproducibility:**

To enhance the reproducibility of your results, we recommend that authors of applicable studies deposit laboratory protocols in protocols.io, where a protocol can be assigned its own identifier (DOI) such that it can be cited independently in the future. Additionally, PLOS ONE offers an option to publish peer-reviewed clinical study protocols. Read more information on sharing protocols at https://plos.org/protocols?utm_medium=editorial-email&utm_source=authorletters&utm_campaign=protocols

Attachment Submitted filename: TSR.docx

Attachment Submitted filename: Review_PCOMPBIOL-D-25-02495.docx

# Author comment: Author response to Decision Letter 1

**DOI:** 10.1371/journal.pcbi.1014385.r002

6 Mar 2026

Attachment Submitted filename: Response to Reviewers.pdf

# Peer review: Decision Letter 1

**DOI:** 10.1371/journal.pcbi.1014385.r003

**Authors:** Lun Hu

14 Apr 2026

PCOMPBIOL-D-25-02495R1

Evolution of phenocopying in a dynamical model of developmental trajectories

PLOS Computational Biology

Dear Dr. Raju,

Thank you for submitting your manuscript to PLOS Computational Biology. After careful consideration, we feel that it has merit but does not fully meet PLOS Computational Biology's publication criteria as it currently stands. Therefore, we invite you to submit a revised version of the manuscript that addresses the points raised during the review process.

Please submit your revised manuscript by Jun 14 2026 11:59PM. If you will need more time than this to complete your revisions, please reply to this message or contact the journal office at ploscompbiol@plos.org. When you're ready to submit your revision, log on to https://www.editorialmanager.com/pcompbiol/ and select the 'Submissions Needing Revision' folder to locate your manuscript file.

Please include the following items when submitting your revised manuscript:

* A letter that responds to each point raised by the editor and reviewer(s). You should upload this letter as a separate file labeled 'Response to Reviewers'. This file does not need to include responses to formatting updates and technical items listed in the 'Journal Requirements' section below.

* A marked-up copy of your manuscript that highlights changes made to the original version. You should upload this as a separate file labeled 'Revised Manuscript with Track Changes'.

* An unmarked version of your revised paper without tracked changes. You should upload this as a separate file labeled 'Manuscript'.

If you would like to make changes to your financial disclosure, competing interests statement, or data availability statement, please make these updates within the submission form at the time of resubmission. Guidelines for resubmitting your figure files are available below the reviewer comments at the end of this letter.

We look forward to receiving your revised manuscript.

Kind regards,

Lun Hu

Academic Editor

PLOS Computational Biology

Natalia Komarova

Section Editor

PLOS Computational Biology

**Additional Editor Comments:**

Although our reviewers acknowledged the improvement of the revised manuscript, they still had some minor concerns regarding this work, such as null-model comparison, claim justification and perturbation design. Due to these issues, I would ask authors to revise their manuscript for another round of revision.

**Reviewers' comments:**

Reviewer's Responses to Questions

**Comments to the Authors:**

**Please note here if the review is uploaded as an attachment.**

Reviewer #1: We suggest that the authors provide clear indications of the changes made and their sources in the response to reviewers. For example, some references suggested in #issue7 are difficult to trace in the revised manuscript. Clearly linking each revision to its corresponding location and citation will greatly facilitate the reviewers’ further evaluation.

Reviewer #3: The revised manuscript is improved and the authors have made a clear effort to address several of the main concerns raised in the first review. In particular, the added clarifications and supplementary analyses strengthen the presentation, but some important issues remain unresolved and a few conclusions still appear stronger than the current evidence supports.

- The null-model comparison is still not controlled tightly enough. The revision adds checks for fitness proximity and sparsity, but the central concern remains because the random functional networks are still not matched to the evolved networks on broader dynamical properties before perturbation. Since the main claim depends on attributing the difference to evolution rather than to residual distributional mismatch between the two ensembles, this part still needs a more controlled comparison or a stronger limitation statement.

- The treatment of large perturbations is still outcome-conditioned, and this remains a major interpretive weakness. The manuscript explicitly keeps only trajectories that are sufficiently far from the base trajectory after perturbation, then analyzes overlap and phenocopy structure on that filtered set. That makes the phenocopy statistics partly conditional on the response itself, which can inflate apparent structure and makes the evolved versus random comparison harder to interpret cleanly. The added explanation helps, but it does not remove the bias introduced by post hoc selection.

- The phenocopy metric is improved but still not fully convincing as a primary quantitative basis for the strongest claims. The authors now add higher-dimensional MDS checks and a distance-matrix-based alternative, which is a useful improvement. Even so, the main text still leans heavily on overlap and localization summaries whose biological interpretation remains indirect. The paper would be stronger if it defined phenocopying in a more direct trajectory-level way and made that the main result rather than a supplementary robustness check.

- The claim that evolution simplifies basin structure is still stronger than the quantitative evidence currently supports. The new attractor-count comparison is helpful, but attractor number alone is not enough to establish basin simplification in a high-dimensional system. The manuscript still relies mainly on projected phase portraits and qualitative basin arguments for this mechanistic conclusion. I would ask the authors to soften this claim or add more direct quantitative basin-level evidence.

-The perturbation design is clearer now, but it also reveals a new conceptual concern. External perturbations are applied to all nodes at once, with no state boundaries, and at many times along the trajectory. That is mathematically permissible, but it makes the environmental perturbation model extremely broad and not especially comparable to localized or structured developmental insults. Because the biological interpretation of phenocopying is one of the paper’s motivations, the manuscript should discuss more explicitly how this global perturbation protocol may shape the observed overlap with internal perturbations.

- The robustness analyses are still somewhat narrow relative to the generality of the claims. The revision adds sensitivity to population size, mutation rate, noise, and endpoint fitness, which is valuable. But the main conclusions are still presented broadly while the explored reference trajectories, perturbation structures, and network settings remain limited. I would ask the authors either to narrow the wording of the main claims or to frame the conclusions more explicitly as applying to this model class rather than to developmental systems in general.

- A new issue is that some of the key validation material appears to have been moved to the supplement while the main paper still draws strong mechanistic conclusions. The revisions rely on supplementary figures for the distance-matrix metric, parameter robustness, attractor counts, and endpoint-fitness comparison. Those additions are important enough that at least one of them, especially the direct non-MDS phenocopy metric, should be promoted into the main text because it directly addresses a central review concern.

**********

**Have the authors made all data and (if applicable) computational code underlying the findings in their manuscript fully available?**

The PLOS Data policy requires authors to make all data and code underlying the findings described in their manuscript fully available without restriction, with rare exception (please refer to the Data Availability Statement in the manuscript PDF file). The data and code should be provided as part of the manuscript or its supporting information, or deposited to a public repository. For example, in addition to summary statistics, the data points behind means, medians and variance measures should be available. If there are restrictions on publicly sharing data or code —e.g. participant privacy or use of data from a third party—those must be specified.

Reviewer #1: None

Reviewer #3: Yes

**********

PLOS authors have the option to publish the peer review history of their article (what does this mean?). If published, this will include your full peer review and any attached files.

If you choose “no”, your identity will remain anonymous but your review may still be made public.

**Do you want your identity to be public for this peer review?**   For information about this choice, including consent withdrawal, please see our Privacy Policy.

Reviewer #1: No

Reviewer #3: No

[NOTE: If reviewer comments were submitted as an attachment file, they will be attached to this email and accessible via the submission site. Please log into your account, locate the manuscript record, and check for the action link "View Attachments". If this link does not appear, there are no attachment files.]

**Figure resubmission:**

-->While revising your submission, we strongly recommend that you use PLOS’s NAAS tool (https://ngplosjournals.pagemajik.ai/artanalysis) to test your figure files. NAAS can convert your figure files to the TIFF file type and meet basic requirements (such as print size, resolution), or provide you with a report on issues that do not meet our requirements and that NAAS cannot fix.

After uploading your figures to PLOS’s NAAS tool - https://ngplosjournals.pagemajik.ai/artanalysis, NAAS will process the files provided and display the results in the "Uploaded Files" section of the page as the processing is complete. If the uploaded figures meet our requirements (or NAAS is able to fix the files to meet our requirements), the figure will be marked as "fixed" above. If NAAS is unable to fix the files, a red "failed" label will appear above. When NAAS has confirmed that the figure files meet our requirements, please download the file via the download option, and include these NAAS processed figure files when submitting your revised manuscript.

**Reproducibility:**

To enhance the reproducibility of your results, we recommend that authors of applicable studies deposit laboratory protocols in protocols.io, where a protocol can be assigned its own identifier (DOI) such that it can be cited independently in the future. Additionally, PLOS ONE offers an option to publish peer-reviewed clinical study protocols. Read more information on sharing protocols at https://plos.org/protocols?utm_medium=editorial-email&utm_source=authorletters&utm_campaign=protocols

# Author comment: Author response to Decision Letter 2

**DOI:** 10.1371/journal.pcbi.1014385.r004

27 Apr 2026

Attachment Submitted filename: Referee Response 2nd Revision.pdf

# Peer review: Decision Letter 2

**DOI:** 10.1371/journal.pcbi.1014385.r005

**Authors:** Lun Hu

19 May 2026

PCOMPBIOL-D-25-02495R2

Evolution of phenocopying in a dynamical model of developmental trajectories

PLOS Computational Biology

Dear Dr. Raju,

Thank you for submitting your manuscript to PLOS Computational Biology. After careful consideration, we feel that it has merit but does not fully meet PLOS Computational Biology's publication criteria as it currently stands. Therefore, we invite you to submit a revised version of the manuscript that addresses the points raised during the review process.

Please submit your revised manuscript by Jul 19 2026 11:59PM. If you will need more time than this to complete your revisions, please reply to this message or contact the journal office at ploscompbiol@plos.org. When you're ready to submit your revision, log on to https://www.editorialmanager.com/pcompbiol/ and select the 'Submissions Needing Revision' folder to locate your manuscript file.

Please include the following items when submitting your revised manuscript:

* A letter that responds to each point raised by the editor and reviewer(s). You should upload this letter as a separate file labeled 'Response to Reviewers'. This file does not need to include responses to formatting updates and technical items listed in the 'Journal Requirements' section below.

* A marked-up copy of your manuscript that highlights changes made to the original version. You should upload this as a separate file labeled 'Revised Manuscript with Track Changes'.

* An unmarked version of your revised paper without tracked changes. You should upload this as a separate file labeled 'Manuscript'.

If you would like to make changes to your financial disclosure, competing interests statement, or data availability statement, please make these updates within the submission form at the time of resubmission. Guidelines for resubmitting your figure files are available below the reviewer comments at the end of this letter.

As the corresponding author, your ORCID iD is verified in the submission system and will appear in the published article. PLOS supports the use of ORCID, and we encourage all coauthors to register for an ORCID iD and use it as well. Please encourage your coauthors to verify their ORCID iD within the submission system before final acceptance, as unverified ORCID iDs will not appear in the published article. Only the individual author can complete the verification step; PLOS staff cannot verify ORCID iDs on behalf of authors.

We look forward to receiving your revised manuscript.

Kind regards,

Natalia L. Komarova

Section Editor

PLOS Computational Biology

Natalia Komarova

Section Editor

PLOS Computational Biology

**Additional Editor Comments :**

One of our reviewers raised several critical concerns regarding the biological interepretation and empirical study. Authors are suggested to clearly state the biological relevance and mechanistic insights of this study.

**********

Note: If the reviewer comments include a recommendation to cite specific previously published works, please review and evaluate these publications to determine whether they are relevant and should be cited. There is no requirement to cite these works unless the editor has indicated otherwise.

**Reviewers' comments:**

Reviewer's Responses to Questions

Reviewer #1: 1. The study relies entirely on computational simulations of developmental trajectories to investigate phenocopying. However, there is no experimental or observational data used to validate the model predictions, which makes it difficult to assess the biological relevance of the results. For high-impact publication, some form of empirical benchmarking or comparison to experimental phenocopy data is recommended.

2. The gene regulatory network model uses idealized ordinary differential equations with uniform random perturbations. These assumptions ignore stochastic effects, epigenetic regulation, and intercellular interactions. The simplifications may limit the model’s ability to capture realistic developmental dynamics, reducing confidence in the conclusions drawn.

3. While the manuscript revisits classical concepts of canalization and robustness, the model largely reproduces known qualitative behaviors without providing new mechanistic insights or predictive power. Figures primarily illustrate trajectory overlaps without rigorous quantitative or biological interpretation, which diminishes the work’s novelty and impact.

Reviewer #3: The authors have addressed the major concerns raised in my previous reviews in a thorough and convincing manner, and these revisions are clearly incorporated throughout the final revised manuscript. Their responses are clear, well organized, and adequately detailed, and the added clarifications and corrections have significantly improved the technical soundness, transparency, and overall consistency of the paper. I did not observe any remaining methodological concerns or major presentation issues that would undermine the validity of the findings or the credibility of the conclusions. At this stage, my recommendations are limited to minor editorial refinements, including careful proofreading for grammar, wording, and stylistic consistency, along with maintaining uniform terminology across the manuscript. Subject to these minor revisions, I consider the manuscript suitable for submission.

**********

**Have the authors made all data and (if applicable) computational code underlying the findings in their manuscript fully available?**

The PLOS Data policy requires authors to make all data and code underlying the findings described in their manuscript fully available without restriction, with rare exception (please refer to the Data Availability Statement in the manuscript PDF file). The data and code should be provided as part of the manuscript or its supporting information, or deposited to a public repository. For example, in addition to summary statistics, the data points behind means, medians and variance measures should be available. If there are restrictions on publicly sharing data or code —e.g. participant privacy or use of data from a third party—those must be specified.

Reviewer #1: None

Reviewer #3: Yes

**********

PLOS authors have the option to publish the peer review history of their article (what does this mean?). If published, this will include your full peer review and any attached files.

If you choose “no”, your identity will remain anonymous but your review may still be made public.

**Do you want your identity to be public for this peer review?**   For information about this choice, including consent withdrawal, please see our Privacy Policy.

Reviewer #1: No

Reviewer #3: No

[NOTE: If reviewer comments were submitted as an attachment file, they will be attached to this email and accessible via the submission site. Please log into your account, locate the manuscript record, and check for the action link "View Attachments". If this link does not appear, there are no attachment files.]

**Figure resubmission:**

-->While revising your submission, we strongly recommend that you use PLOS’s NAAS tool (https://ngplosjournals.pagemajik.ai/artanalysis) to test your figure files. NAAS can convert your figure files to the TIFF file type and meet basic requirements (such as print size, resolution), or provide you with a report on issues that do not meet our requirements and that NAAS cannot fix.

After uploading your figures to PLOS’s NAAS tool - https://ngplosjournals.pagemajik.ai/artanalysis, NAAS will process the files provided and display the results in the "Uploaded Files" section of the page as the processing is complete. If the uploaded figures meet our requirements (or NAAS is able to fix the files to meet our requirements), the figure will be marked as "fixed" above. If NAAS is unable to fix the files, a red "failed" label will appear above. When NAAS has confirmed that the figure files meet our requirements, please download the file via the download option, and include these NAAS processed figure files when submitting your revised manuscript.

**Reproducibility:**

To enhance the reproducibility of your results, we recommend that authors of applicable studies deposit laboratory protocols in protocols.io, where a protocol can be assigned its own identifier (DOI) such that it can be cited independently in the future. Additionally, PLOS ONE offers an option to publish peer-reviewed clinical study protocols. Read more information on sharing protocols at https://plos.org/protocols?utm_medium=editorial-email&utm_source=authorletters&utm_campaign=protocols

# Author comment: Author response to Decision Letter 3

**DOI:** 10.1371/journal.pcbi.1014385.r006

21 May 2026

Attachment Submitted filename: Reviewer Response PLOS Comp Bio Resubmission 3.pdf

# Editor report: Decision Letter 3

**DOI:** 10.1371/journal.pcbi.1014385.r007

**Authors:** Lun Hu

1 Jun 2026

Dear Dr. Raju,

We are pleased to inform you that your manuscript 'Evolution of phenocopying in a dynamical model of developmental trajectories' has been provisionally accepted for publication in PLOS Computational Biology.

Before your manuscript can be formally accepted you will need to complete some formatting changes, which you will receive in a follow up email. A member of our team will be in touch with a set of requests.

Please note that your manuscript will not be scheduled for publication until you have made the required changes, so a swift response is appreciated.

IMPORTANT: The editorial review process is now complete. PLOS will only permit corrections to spelling, formatting or significant scientific errors from this point onwards. Requests for major changes, or any which affect the scientific understanding of your work, will cause delays to the publication date of your manuscript.

Should you, your institution's press office or the journal office choose to press release your paper, you will automatically be opted out of early publication. We ask that you notify us now if you or your institution is planning to press release the article. All press must be co-ordinated with PLOS.

Thank you again for supporting Open Access publishing; we are looking forward to publishing your work in PLOS Computational Biology.

Best regards,

Lun Hu

Academic Editor

PLOS Computational Biology

Natalia Komarova

Section Editor

PLOS Computational Biology

***********************************************************

The concerns of all reviewers have been addressed in this revised manuscript, and therefore I recommend to accept tihs work as a regular paper.

# Editor report: Acceptance letter

**DOI:** 10.1371/journal.pcbi.1014385.r008

**Authors:** Lun Hu

PCOMPBIOL-D-25-02495R3

Evolution of phenocopying in a dynamical model of developmental trajectories

Dear Dr Raju,

I am pleased to inform you that your manuscript has been formally accepted for publication in PLOS Computational Biology. Your manuscript is now with our production department and you will be notified of the publication date in due course.

The corresponding author will soon be receiving a typeset proof for review, to ensure errors have not been introduced during production. Please review the PDF proof of your manuscript carefully, as this is the last chance to correct any errors. Please note that major changes, or those which affect the scientific understanding of the work, will likely cause delays to the publication date of your manuscript.

Soon after your final files are uploaded, unless you have opted out, the early version of your manuscript will be published online. The date of the early version will be your article's publication date. The final article will be published to the same URL, and all versions of the paper will be accessible to readers.

For Research, Software, and Methods articles, you will receive an invoice from PLOS for your publication fee after your manuscript has reached the completed accept phase. If you receive an email requesting payment before acceptance or for any other service, this may be a phishing scheme. Learn how to identify phishing emails and protect your accounts at https://explore.plos.org/phishing.

Thank you again for supporting PLOS Computational Biology and open-access publishing. We are looking forward to publishing your work!

With kind regards,

Anita Estes

PLOS Computational Biology | Carlyle House, Carlyle Road, Cambridge CB4 3DN | United Kingdom ploscompbiol@plos.org | Phone +44 (0) 1223-442824 | ploscompbiol.org | @PLOSCompBiol
